# Phenotypic analysis of *Myo10* knockout (*Myo10*^tm2/tm2^) mice lacking full-length (motorized) but not brain-specific headless myosin X

**DOI:** 10.1038/s41598-018-37160-y

**Published:** 2019-01-24

**Authors:** Anne C. Bachg, Markus Horsthemke, Boris V. Skryabin, Tim Klasen, Nina Nagelmann, Cornelius Faber, Emma Woodham, Laura M. Machesky, Sandra Bachg, Richard Stange, Hyun-Woo Jeong, Ralf H. Adams, Martin Bähler, Peter J. Hanley

**Affiliations:** 1 Institut für Molekulare Zellbiologie, Westfälische Wilhelms-Universität Münster, 48149 Münster, Germany; 2 Department of Medicine, Transgenic Animal and Genetic Engineering Models (TRAM), Westfälische Wilhelms-Universität Münster, 48149 Münster, Germany; 3 Department of Clinical Radiology, Westfälische Wilhelms-Universität Münster, 48149 Münster, Germany; 4 Cancer Research UK Beatson Institute, Glasgow University College of Medical, Veterinary and Life Sciences Garscube Estate, Glasgow, G61 1BD United Kingdom; 5 Department of Regenerative Musculoskeletal Medicine, Institute of Musculoskeletal Medicine (IMM), University Hospital Münster, 48149 Münster, Germany; 6 Max Planck Institute for Molecular Biomedicine, Department of Tissue Morphogenesis, and University of Münster, Faculty of Medicine, 48149 Münster, Germany

## Abstract

We investigated the physiological functions of Myo10 (myosin X) using *Myo10* reporter knockout (*Myo10*^tm2^) mice. Full-length (motorized) Myo10 protein was deleted, but the brain-specific headless (Hdl) isoform (Hdl-Myo10) was still expressed in homozygous mutants. *In vitro*, we confirmed that Hdl-Myo10 does not induce filopodia, but it strongly localized to the plasma membrane independent of the MyTH4-FERM domain. Filopodia-inducing Myo10 is implicated in axon guidance and mice lacking the Myo10 cargo protein DCC (deleted in colorectal cancer) have severe commissural defects, whereas MRI (magnetic resonance imaging) of isolated brains revealed intact commissures in Myo10^tm2/tm2^ mice. However, reminiscent of Waardenburg syndrome, a neural crest disorder, *Myo10*^tm2/tm2^ mice exhibited pigmentation defects (white belly spots) and simple syndactyly with high penetrance (>95%), and 24% of mutant embryos developed exencephalus, a neural tube closure defect. Furthermore, *Myo10*^tm2/tm2^ mice consistently displayed bilateral persistence of the hyaloid vasculature, revealed by MRI and retinal whole-mount preparations. In principle, impaired tissue clearance could contribute to persistence of hyaloid vasculature and syndactyly. However, Myo10-deficient macrophages exhibited no defects in the phagocytosis of apoptotic or IgG-opsonized cells. RNA sequence analysis showed that *Myo10* was the most strongly expressed unconventional myosin in retinal vascular endothelial cells and expression levels increased 4-fold between P6 and P15, when vertical sprouting angiogenesis gives rise to deeper layers. Nevertheless, imaging of isolated adult mutant retinas did not reveal vascularization defects. In summary, Myo10 is important for both prenatal (neural tube closure and digit formation) and postnatal development (hyaloid regression, but not retinal vascularization).

## Introduction

Unconventional myosins expressed in humans and mice are divided into classes (I, III, V, VI, VII, IX, X, XV, XVI, XVIII and XIX), and individual myosins typically exhibit class-specific functions. For example, class V myosins have been implicated in vesicular trafficking^1^ and class IX myosins are RhoGAPs (Rho GTPase-activating proteins)^2^. However, the function of myosin X (Myo10), the only class X member, is largely unknown. It belongs to the group of MyTH4-FERM (myosin tail homology 4 - band 4.1, ezrin, radixin, moesin) myosins, which includes classes VII and XV. MyTH4-FERM myosins localize to structures containing bundled actin, such as filopodia (Myo10)^3,4^, stereocilia (Myo7a and Myo15)^5,6^ and microvilli (Myo7b)^7,8^. The MyTH4-FERM domains of class VII myosins are implicated in linking actin to cadherins, through adaptor proteins, which provide linkages between adjacent stereocilia (Myo7a) and microvilli (Myo7b)^9^. Myo15 is important for elongation of stereocilia and heterologous expression of GFP-tagged Myo15 induces filopodia formation^10^. Notably, mutations of *Myo7a* or *Myo15* (human ortholog *MYO15A*) cause deafness^11^. The MyTH4-FERM domain of Myo10 may facilitate filopodium-cell or filopodium-ECM (extracellular matrix) adhesion by binding cadherins^12,13^, β-integrins^14^ or DCC (deleted in colorectal cancer)^15^. Heterologous expression of Myo10 robustly induces filopodia formation, the most conspicuous function of Myo10, and the motor protein localizes to the tips of these structures^16^. Structural studies indicated that Myo10 dimerizes antiparallel via its CC (coiled-coil) domain^17^, which may allow the motor protein to crosslink and move along parallel actin tracks. In addition to MyTH4-FERM and CC domains, Myo10 contains PH (pleckstrin homology) domains, which confer binding to membrane phosphoinositides^18^.

The physiological functions of the MyTH4-FERM- and PH domain-containing protein Myo10 are not clear. Since the first detailed description of full-length Myo10 in 2000^19^, Myo10 has been implicated in diverse functions, including spindle assembly^20^, endothelial tube formation^21^, melanosome transfer^22^ (which involves filopodia formation), axon guidance^23^, cell motility^24,25^ and phagocytosis^26^. Here, we report the phenotypes of *Myo10* reporter knockout mice, which turn out to lack full-length (motorized) Myo10, but still express the brain-specifc, headless isoform. While preparing this manuscript, the phenotypes of Myo10^tm1d/tm1d^ mice^27^, which lack both full-length and headless Myo10, as well as Myo10^tm2/tm2^ mice^28^, the mutant strain used in this study, were reported.

## Results

### *Myo10* reporter knockout mice

**Figure 1:**
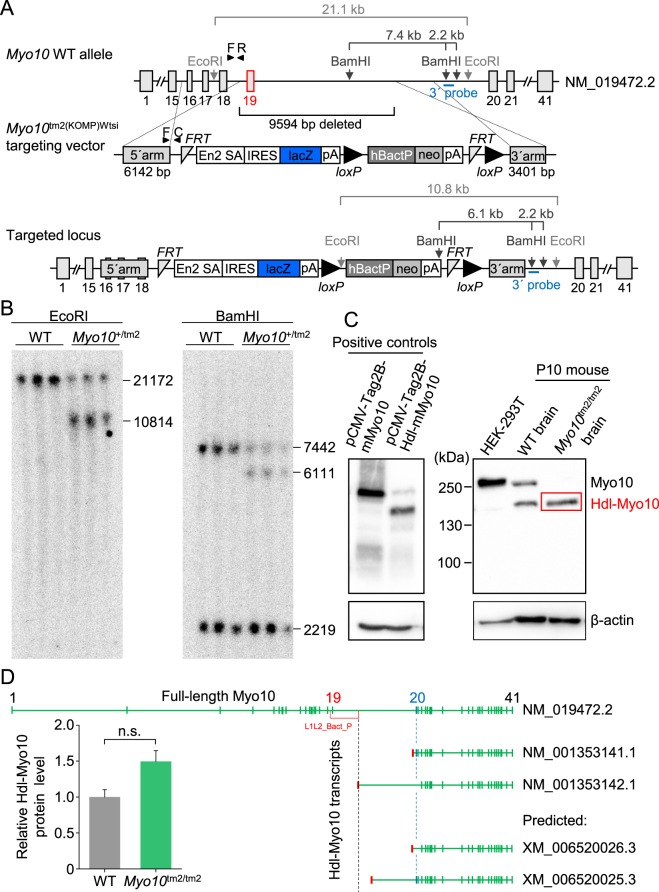
*Myo10* reporter knockout (*Myo10*^tm2/tm2^) mice lack full-length (motorized) Myo10, but express the brain-specific headless isoform. (**A**) Schematic diagram showing the reporter knockout (tm2) targeting strategy. Insertion of the targeting sequence by homologous recombination causes loss of 9594 bp, including exon 19 and part of intron 19. Notably, the headless Myo10 isoform begins at exon 20. The gene trap, polyadenylation (pA) signal, is harbored in the IRES:lacZ cassette (IRES stands for internal ribosome entry site). (**B**) Southern blot analysis. DNA was fragmented using the restriction enzyme EcoRI or BamHI. The position of the radiolabeled hybridization probe at the 3′-end is indicated. Labeled DNA fragments were detected using X-ray film. (**C**) Western blot analysis. Lysates of HEK293T cells overexpressing full-length mouse (m) Myo10 (plasmid, pCMV-Tag2B-mMyo10) or headless (Hdl) mouse Myo10 (plasmid, pCMV-Tag2B-Hdl-mMyo10) were used as positive controls (blot on the left). Whole brain lysates obtained from P10 mice were used to screen for expression of full-length and Hdl-Myo10 (blot on the right). (**D**) Level of Hdl-Myo10 protein expressed in *Myo10*^tm2/tm2^ mouse brain (n = 3) relative to wild-type (WT) brains (n = 3), and mouse Hdl-Myo10 transcripts obtained from the National Center for Biotechnology Information (NCBI), National Institutes of Health (NIH). The accession prefix NM_ denotes confirmed protein-coding transcripts, whereas the prefix XM_ indicates predicted protein transcripts. Green (vertical) bars are exons (ranging from 1 to 41; indicated above) and red bars are 5′-UTRs (5′ untranslated regions), preceding the coding sequence. Notably, the 5′-UTR of Hdl-Myo10 transcript NM_001353142.1, which lacks exon 20 (labeled blue) and exon 21, is disrupted by insertion of the cassette (L1L2_Bact_P) used to generate the *Myo10*^tm2^ allele.

The reporter knockout (tm2) targeting strategy for *Myo10* is shown in Fig. 1A. Insertion of the targeting cassette causes deletion of exon 19 and part of intron 19, and introduces both a reporter (*lacZ* gene) of endogenous gene expression and a gene trap (SV40 (simian virus 40) polyadenylation (pA) signal). Notably, the mutant (*Myo10*^tm2^) allele cannot be converted to a conditional allele. Instead, Cre recombination would delete the *loxP*-flanked selection cassette, whereas Flp recombination would produce an exon 19 deletion allele without reading frame shift. Southern blot analysis using the hybridization probe shown in Fig. 1A confirmed correct targeting (Fig. 1B). Western blot analysis confirmed that full-length Myo10 was deleted in mouse postnatal (P10) brain (Fig. 1C). However, the headless (Hdl) isoform of Myo10 (Hdl-Myo10) could be clearly detected in homozygous mutants. Notably, lysates from HEK293T cells overexpressing full-length mouse Myo10 (mMyo10) or mouse headless Myo10 (Hdl-mMyo10) were used as positive controls for the anti-Myo10 antibody (Fig. 1C). On the one hand, lack of Hdl-Myo10 deletion in mutant mice is not completely surprising since the transcript for Hdl-Myo10 is downstream from exon 19^29^. On the other hand, as alluded to previously^27^, the 5′ untranslated region (5′-UTR) of one of the two confirmed transcripts (NM_001353141.1 and NM_001353142.1) corresponding to Hdl-Myo10 is predicted to be disrupted in the *Myo10*^tm2^ allele (Fig. 1D). The affected transcript (NM_001353142.1) lacks exons 20 and 21, suggesting that it may encode a minor isoform of Hdl-Myo10^30^ (Fig. 1D). In any case, Western blot analysis revealed no significant difference in relative mouse brain Hdl-Myo10 levels: blot densities (Fig. 1D). Thus, the Hdl-Myo10 isoform encoded by NM_001353141.1 may be the major Hdl-Myo10 isoform in mouse brain or it may be upregulated in response to loss of the transcript NM_001353142.1 in the *Myo10*^tm2^ allele.

### Viability of homozygous *Myo10* reporter knockout (*Myo10*^tm2/tm2^) mice

**Figure 2:**
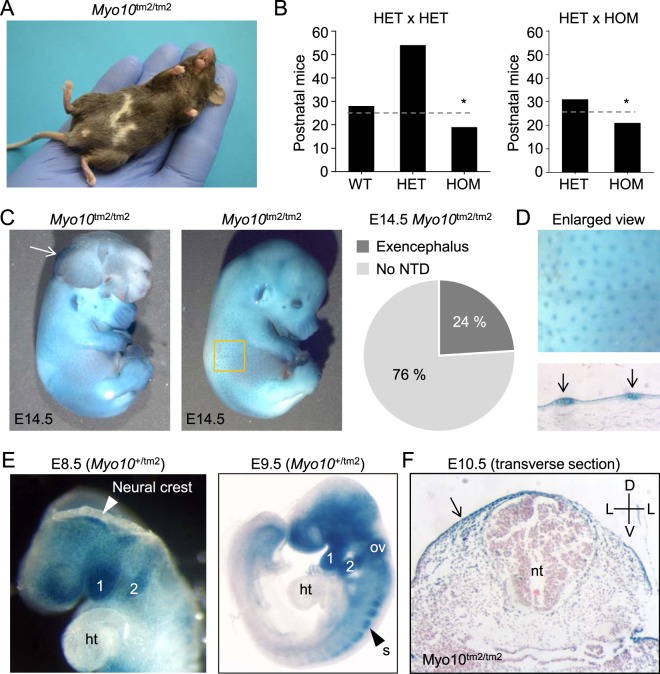
Homozygous *Myo10* reporter knockout (*Myo10*^tm2/tm2^) mouse embryos develop exencephaly. (**A**) Pigmentation defects in homozygous mutants. Image of an adult *Myo10*^tm2/tm2^ mouse showing white belly spots. (**B**) Genotype frequency of offspring derived from HET (heterozygous) x HET and HET x HOM (homozygous) matings. HOM offspring were produced at less than expected frequency, indicated by dashed lines and asterices. (**C**) Two examples of X-gal stained, homozygous *Myo10* mutant embryos at E14.5, with (left) and without (right) exencephalus, caused by failure of the cranial neural tube to close. The white arrow on the left indicates everted cranial neural folds, a hallmark of exencephalus. About 1 in 4 (24%) of homozygous *Myo10* mutant (*Myo10*^tm2/tm2^) embryos developed exencephalus. Neural tube defect is abbreviated NTD. (**D**) Enlarged view from panel C (yellow square) and skin histological section showing *Myo10* expression in the skin and hair placodes (blue spots). (**E**) Whole-mount X-gal staining. *Myo10* is expressed in the head and the first and second branchial arches (labeled 1 and 2, respectively) of the developing embryo (E8.5 and E9.5). (**F**) X-gal staining and histology (E10.5) reveals expression of *Myo10* in the ectoderm and dorsal regions, but not in the neural tube. ht, heart; ov, otic vesicle; s, somite; nt, neural tube; D, dorsal; V, ventral; L, lateral.

Homozygous, but not heterozygous, *Myo10* reporter knockout mice consistently exhibited pigmentation defects, white belly spots (Fig. 2A). Otherwise, homozygous mutants appeared healthy and fertile. However, mating of heterozygous (HET) mice (HET × HET) or heterozygous and homozygous (HOM) mice (HET × HOM) produced less homozygous mutant mice than expected by Mendelian inheritance (Fig. 2B). This discrepancy could be explained by the development of exencephalus, a neural tube closure defect, in 24% of *Myo10*^tm2/tm2^ embryos (Fig. 2C). Failure of the neural tube to close causes the neuroepithelium to protrude (exencephaly), which through degeneration progresses to anencephaly^31^.

Whole-mount E14.5 *Myo10*^tm2/tm2^ (Fig. 2C) and *Myo10*^+/tm2^ (not shown for E14.5) embryos were stained with X-gal to visualize *Myo10* expression (X-gal becomes intensely blue following cleavage by β-galactosidase, the enzyme encoded by the reporter gene *lacZ*). *Myo10* expression (X-gal staining) could be clearly detected in the developing skin and hair placodes (Fig. 2D). At E8.5 - E9.5, *Myo10* was expressed in the first and second branchial arches, as well as in the otic vesicle and somites (Fig. 2E). *Myo10* was not detected in the heart (E9.5). Transverse sections of a paraffin embedded and X-gal stained E10.5 *Myo10*^tm2/tm2^ embryo revealed *Myo10* expression in the developing epidermis and dorsolaterally in the dermis (Fig. 2F).

### Headless *Myo10* localizes to the plasma membrane independent of the MyTH4-FERM domain

**Figure 3:**
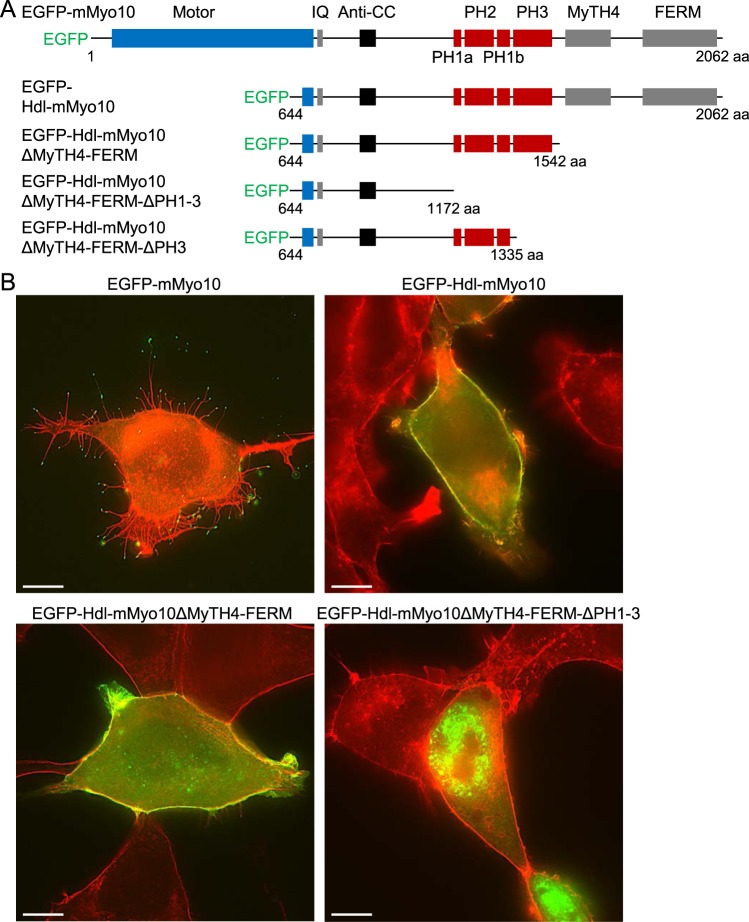
Headless mouse Myo10 (Hdl-mMyo10) strongly localizes to the cell periphery independent of the MyTH4-FERM domain. (**A**) Schematic representations of the domain structures of N-terminal EGFP-tagged mouse Myo10 (EGFP-mMyo10), Hdl-mMyo10 (EGFP- Hdl-mMyo10) and truncated variants thereof. (**B**) Images of fixed HEK293T cells after transfection with EGFP-mMyo10 or various EGFP-tagged Hdl-mMyo10 constructs. Cells were counterstained with Alexa Fluor 594-conjugated phalloidin, a red fluorescent F-actin probe, and imaged via a Zeiss Plan Apo 63/1.4 (oil-immersion) objective lens by superresolution structured illumination microscopy. Scale bars: 10 µm.

**Figure 4:**
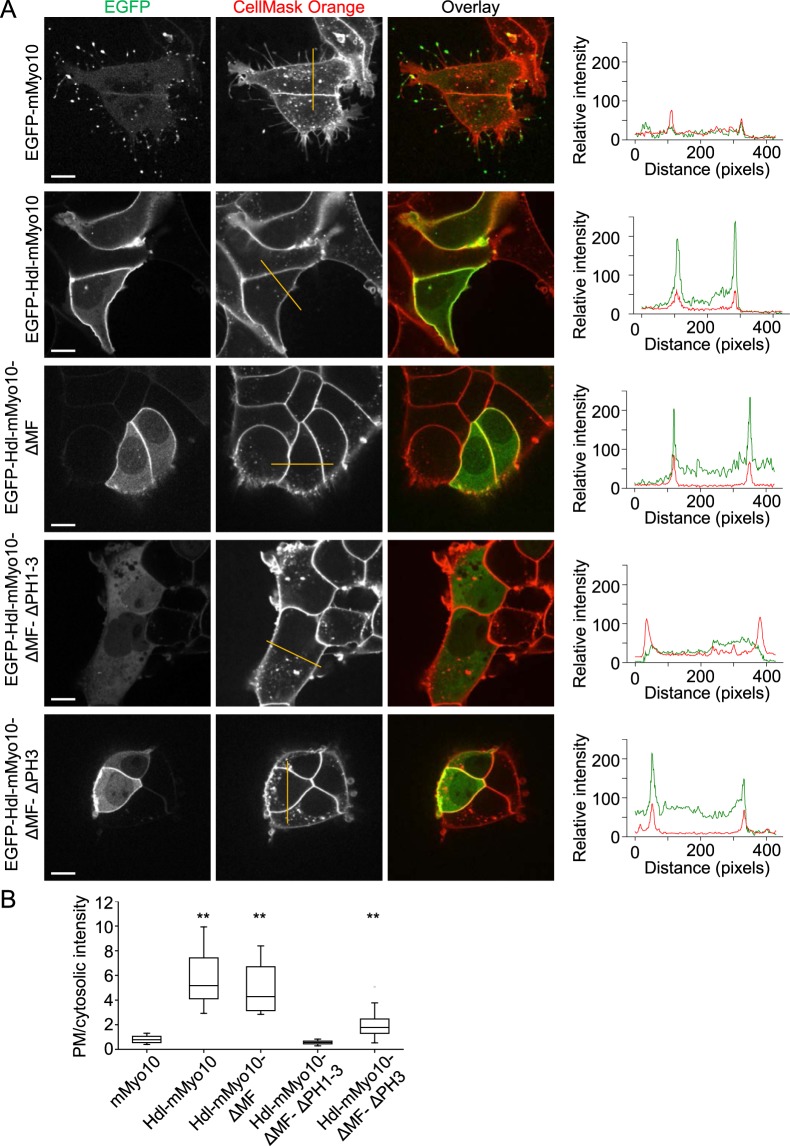
Localization of headless Myo10 to the plasma membrane requires pleckstrin homology (PH) domains. (**A**) Fluorescence images of living HEK293T cells transfected with EGFP-tagged, full-length mouse Myo10 (mMyo10-EGFP) or various EGFP-tagged headless mouse Myo10 (Hdl-mMyo10) constructs. Cells were counterstained with the red fluorescent plasma membrane marker CellMask Orange. Images were obtained by spinning disk confocal microscopy via a Nikon Apochromat TIRF 60x/1.49 (oil-immersion) objective lens. Scale bars: 10 µm. Plots of gray value intensity for the superimposed lines (dark yellow lines in the middle column) are shown on the right. In the plots of intensity along the lines, peaks of the CellMask Orange (red) traces serve as plasma membrane markers. (**B**) Profile data quantification. Plot of relative plasma membrane localization, indexed as the ratio of the maximum of the plasma membrane (PM) peak to mean cytosolic intensity.

The domain structures of the mouse Myo10 (mMyo10) and EGFP-tagged truncation constructs used to explore the subcellular localization of headless Myo10 (Hdl-mMyo10) are shown in Fig. 3A. Cells were fixed, stained with Alexa Fluor 594-conjugated phalloidin (an F-actin probe) and imaged by superresolution structured illumination microscopy. As expected from earlier work^29,30^, transfection of HEK293T cells with full-length EGFP-tagged mouse Myo10 (EGFP-mMyo10) induced filopodia formation, whereas transfection with EGFP-Hdl-mMyo10 failed to induce filopodia (Fig. 3B). However, EGFP-Hdl-mMyo10 impressively localized to the plasma membrane suggesting that the tail PH domains readily recruits the protein to membrane phosphoinositides, possibly due to loss of head-tail autoinhibition. Consistent with this notion, deletion of the MyTH4-FERM domain had no effect, whereas deletion of the PH domains completely blocked membrane localization (Fig. 3B). In living cells stained with the fluorescent plasma membrane probe CellMask Orange and transfected with various deletion constructs, we confirmed using quantified linear profile plots that EGFP-Hdl-mMyo10 and Myo10 lacking the MyTH4-FERM domain (EGFP-mMyo10-ΔMF) strongly localized to the plasma membrane, whereas Myo10 lacking PH domains (EGFP-Hdl-mMyo10-ΔMF-ΔPH1-3) did not localize to the membrane (Fig. 4A,B). Furthermore, deletion of one of the PH domains (EGFP-Hdl-mMyo10-ΔMF-ΔPH3) reduced membrane localization (Fig. 4A,B). Thus, Hdl-Myo10 is probably strongly recruited to the membrane due to the absence of head-tail inhibition. This mechanism would complement the head-tail interaction model proposed by Umeki *et al*.^32^ in which phospholipid binding to Myo10 disrupts head-tail interactions and promotes dimerization, converting the myosin into a filopodial cargo transporter. Head-tail interactions, otherwise, maintain the inactive folded conformation.

### *Myo10*^tm2/tm2^ mice do not phenocopy *Dcc* and *Ntn1* knockout mouse models

**Figure 5:**
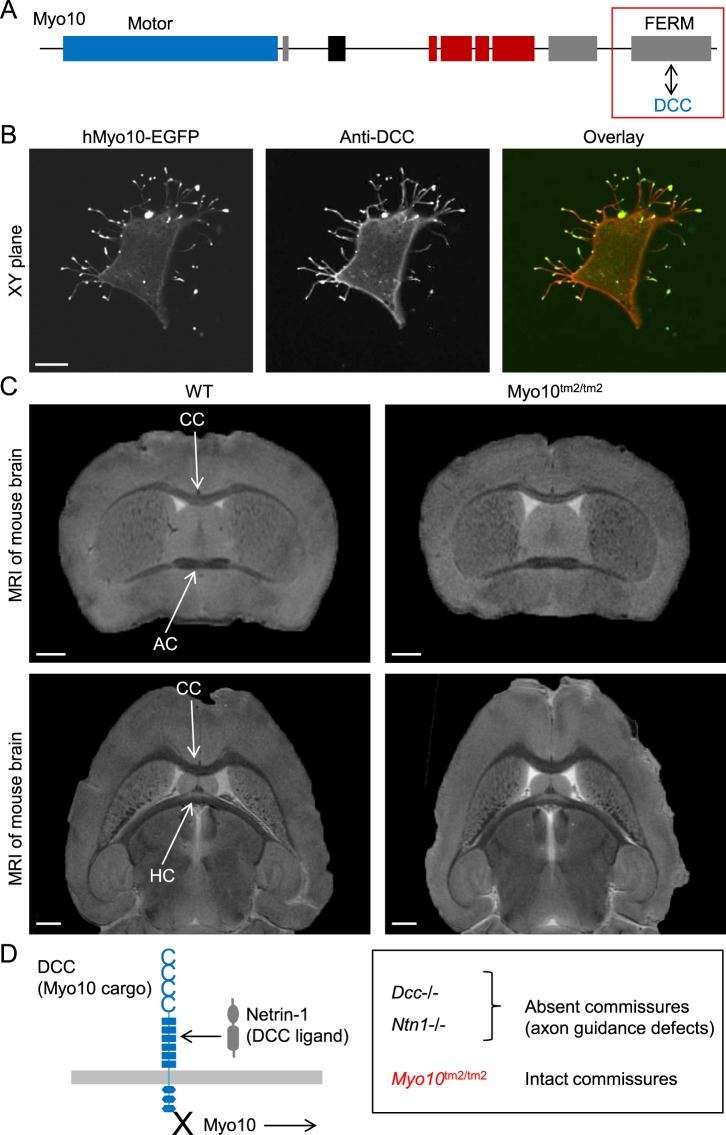
Mice lacking full-length Myo10 have intact brain commissures. (**A**) Schematic diagram illustrating the interaction of the cargo protein DCC (deleted in colorectal cancer) with the cargo-binding FERM domain of Myo10. (**B**) Colocalization of DCC and Myo10 to the tips of filopodia. HEK293T cells were transfected with human DCC and EGFP-tagged human Myo10 (hMyo10-EGFP), and subsequently fixed and labeled with anti-DCC antibodies which recognize an extracellular domain. Scale bar: 10 µm. (**C**) Coronal and horizontal sections of isolated and fixed mouse brains obtained by MRI (magnetic resonance imaging). Scale bars: 1 mm. CC, corpus callosum; AC, anterior commissure; HC, hippocampal commissure. (**D**) Schematic summary diagram. Mice lacking DCC (encoded by *Dcc*) or the DCC ligand Netrin-1 (encoded by *Ntn1*) have absent or negligible commissures, whereas commissures are intact in mice lacking the DCC transport protein Myo10.

DCC (encoded by *Dcc*), a member of the immunoglobulin superfamily of cell adhesion molecules, is involved in the guidance of axons towards sources of the ligand Netrin-1, encoded by *Ntn1*. Notably, filopodia are important components of growth cones and the axon guidance machinery^33^. In mice lacking either the transmembrane protein DCC^34^ or its ligand Netrin-1^35^, the corpus callosum and hippocampal commissure appear to be absent, and the anterior commissure is negligible^34,35^. In structural studies, the MyTH4-FERM domain of Myo10 has been shown to bind the cytosolic tail domain of DCC^15,36^, a cargo protein, as schematically shown in Fig. 5A. We confirmed that DCC localized to the tips of Myo10-induced filopodia in HEK293T cells transfected with both human DCC and EGFP-tagged human Myo10 (Fig. 5B). DCC was labeled with mouse monoclonal antibodies which recognize the extracellular domain of human DCC.

Using high-resolution MRI (magnetic resonance imaging), we investigated whether *Myo10*^tm2/tm2^ mice had defects in the commissures of the brain (Fig. 5C). MRI of fixed brains isolated from WT and *Myo10*^tm2/tm2^ mice revealed that full-length Myo10 is not critical for the formation of the corpus callosum, anterior commissure and hippocampal commissure (Fig. 5C), in contrast to *Dcc* (DCC) and *Ntn1* (Netrin-1) knockout mice^34,35^, as summarized in Fig. 5D. In coronal views, for example, the corpus callosum had a mean midline thickness of 0.28 ± 0.02 mm in both WT and *Myo10*^tm2/tm2^ brains (n = 3 for each group).

### White belly spots are devoid of melanocytes

**Figure 6:**
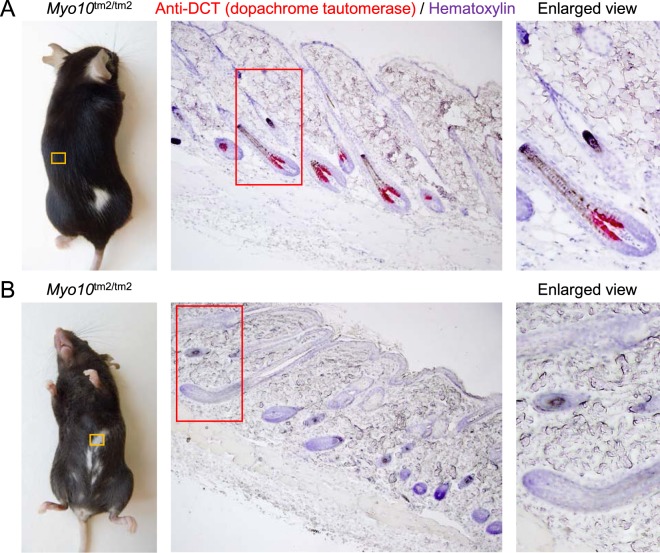
White belly spots of *Myo10*^tm2/tm2^ mice are devoid of melanocytes. (**A**) Histological section of dorsal skin containing dark hair taken from a *Myo10*^tm2/tm2^ mouse and stained with anti-DCT antibodies (red; DCT (dopachrome tautomerase) is a melanocyte marker) and hematoxylin (blue). (**B**) Section of skin taken from a white belly spot of a *Myo10*^tm2/tm2^ mouse and stained with anti-DCT antibodies and hematoxylin.

Myo10 has been implicated in the transfer of melanosomes from melanocytes into epidermal keratinocytes^22^. If Myo10 was important for melanosome transfer, deletion of full-length Myo10 would be expected to produce a coat color phenotype similar to Myo5a-deficient mice^37^, which have widespread hypopigmentation. Instead, *Myo10*^tm2/tm2^ mice have white belly patches, useful as an indicator of the homozygous genotype (Fig. 2A), and infrequently a dorsal white patch (Fig. 6A). We confirmed using histological skin sections and antibodies against DCT (dopachrome tautomerase), a melanocyte marker, that the hair follicles in white patches were not populated with melanocytes (Fig. 6B).

### Phenotype of syndactyly in *Myo10*^tm2/tm2^ mice

**Figure 7:**
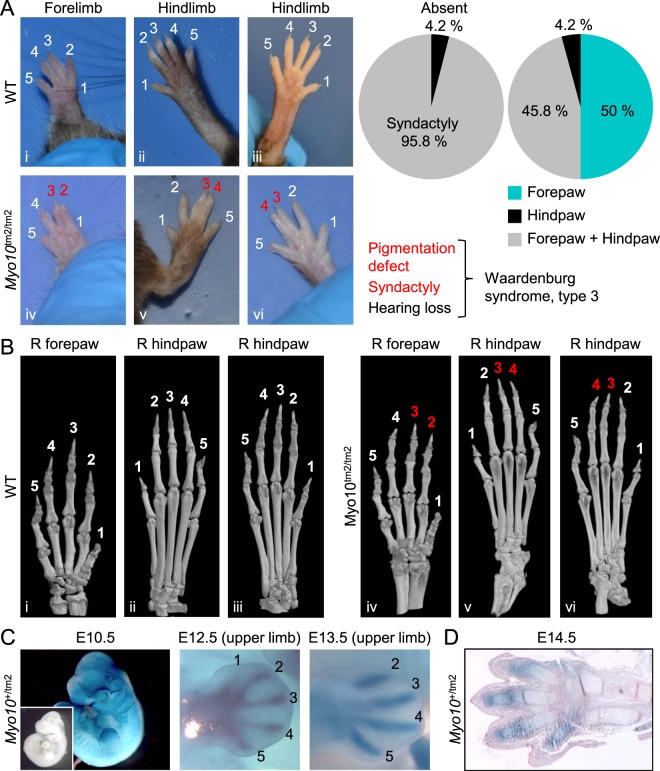
High penetrance of simple syndactyly in *Myo10*^tm2/tm2^ mice. (**A**) Examples of normal paws in WT mice (top panel) and syndactyly in *Myo10*^tm2/tm2^ mice (lower panel). Typically, digits 2 and 3 or digits 3 and 4 were fused. The frequency (n = 25 mice) and variations (assessed in n = 24 mice) of the phenotype (simple syndactyly) are shown in the pie charts. As indicated below the pie charts, the unusual combination of pigmentation defects and syndactyly has been sporadically reported in cases of Waardenburg syndrome, type 3, which usually includes loss of hearing (not tested). (**B**) Three dimensional (3D) micro-computed tomography (µCT) images of mouse paws. The µCT images were obtained from the same paws shown above (Roman numerals indicate the matching images). (**C**) X-gal staining of whole-mount *Myo10*^+/tm2^ embryos showing *Myo10* expression in the developing limb bud (E10.5) and digit primordia (E12.5 and E13.5). The inset at E10.5 shows an X-gal stained (control) WT embryo. (**D**) Histological section of an X-gal stained autopod (and distal zeugopod) at E14.5. The section was counterstained with eosin (pink).

Mice lacking full-length Myo10 exhibited simple (soft tissue) syndactyly with high penetrance (Fig. 7A). Typically, digits 2 and 3 and/or digits 3 and 4 were completely fused (frequency of 31: 7: 1 for fusion of digits 2/3, 3/4 and 2/3/4, respectively; n = 14 mice). The rare combination of syndactyly and pigmentation defects in *Myo10*^tm2/tm2^ mice is reminiscent of variations of Waardenburg syndrome (type 3), a neural crest cell disorder which is usually associated with hearing loss^38,39^. We confirmed using µCT (micro-computed tomography) that the syndactyly phenotype of *Myo10*^tm2/tm2^ mice did not involve osseous fusion (Fig. 7B; the µCT scans shown in Fig. 7B correspond to the photographed paws shown in Fig. 7A and can be matched by the Roman numerals in the lower left corner of each image).

X-gal staining of E10.5 - E13.5 embryos revealed *Myo10* expression in the developing limb bud and digit primordia (Fig. 7C). At E12.5 and E13.5, there was clear X-gal staining (*Myo10* expression) of autopod condensations (Fig. 7C). Histological sections of the footplate at E14.5, when digit separation was nearly complete, revealed X-gal staining in the developing perichondrium and joints (Fig. 7D).

### Persistence of hyaloid vasculature

**Figure 8:**
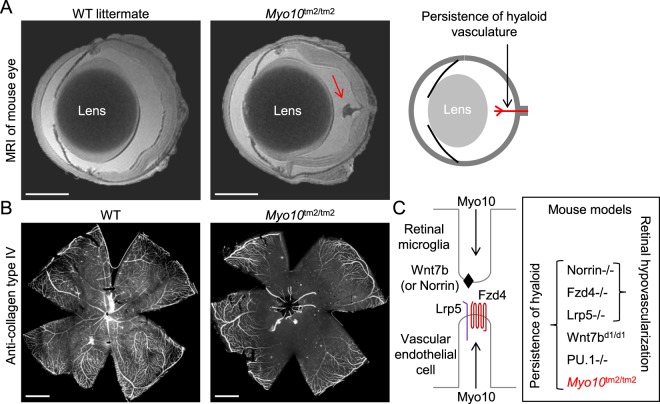
High penetrance of persistence of the hyaloid vasculature, without retinal hypovascularization, in *Myo10*^tm2/tm2^ mice. (**A**) High-resolution MRI (magnetic resonance imaging) of enucleated and fixed eyes from adult wild-type (WT) and *Myo10*^tm2/tm2^ mice (representative of 6 eye scans for each genotype) reveals persistence of the hyaloid vasculature in mutant mice. The hyaloid artery emerges from the optic disc and extends towards the lens, as schematically illustrated on the right. Scale bars: 1 mm. (**B**) Whole-mount retina from an adult WT and *Myo10*^tm2/tm2^ mouse stained with anti-collagen, type IV antibodies. Hyaloid vessels can be seen emerging from the central optic disc of the *Myo10*^tm2/tm2^ retina. Scale bar: 1 mm. (**C**) Schematic diagram showing putative roles of Myo10 in macrophage-to-endothelial cell Wnt signaling, and knockout mouse models in which hyaloid persistence phenotypes, with or without retinal hypovascularization, manifest. Notably, Wnt7b^d1^ is a hypomorphic allele, implying that there is reduced, but not absent, Wnt7b gene function in Wnt7b^d1/d1^ mice. PU.1 null (PU.1−/−) mice lack macrophages.

The hyaloid vasculature extends from the optic disc and spans the vitreous to supply blood to the growing lens. In mouse, this vasculature is extensive in the first few postnatal days, but almost completely regresses in the following 2–3 weeks, with marked regression already apparent at postnatal day 8 (P8)^40^. High-resolution MRI of fixed enucleated eyes revealed that the hyaloid vasculature persists in adult *Myo10*^tm2/tm2^ mice (Fig. 8A). Persistent hyaloid vessels could also be seen in retinal whole-mount preparations labeled with anti-collagen, type IV antibodies (Fig. 8B). Lobov *et al*.^41^. elegantly deduced that regression of the hyaloid vasculature is initiated by macrophages and requires Wnt7b signaling from macrophages to target vascular endothelial cells, which express the Wnt receptor Fzd4 (frizzled class receptor 4) and its co-receptor Lrp5 (low density lipoprotein receptor-related protein 5). Notably, mice lacking the receptor Fzd4, the co-receptor Lrp5 or the ligands Wnt7b or Norrin exhibit persistence of the hyaloid vasculature^41–44^, schematically illustrated in Fig. 8C. To test whether overexpression of Myo10 enriches Fzd4 at the tips of filopodia, we cotransfected HEK293T cells with EGFP-tagged mouse Fzd4 (Fzd4-EGFP) and mCherry-tagged bovine Myo10 (mCherry-bMyo10). Fzd4-EGFP localized to the plasma membrane and filopodia, but it was not enriched at filopodial tips (not shown). In contrast, HEK293T cells transfected with TurboGFP-tagged human Wnt7b (Wnt7b-tGFP) did not exhibit plasma membrane labeling (not shown).

**Figure 9:**
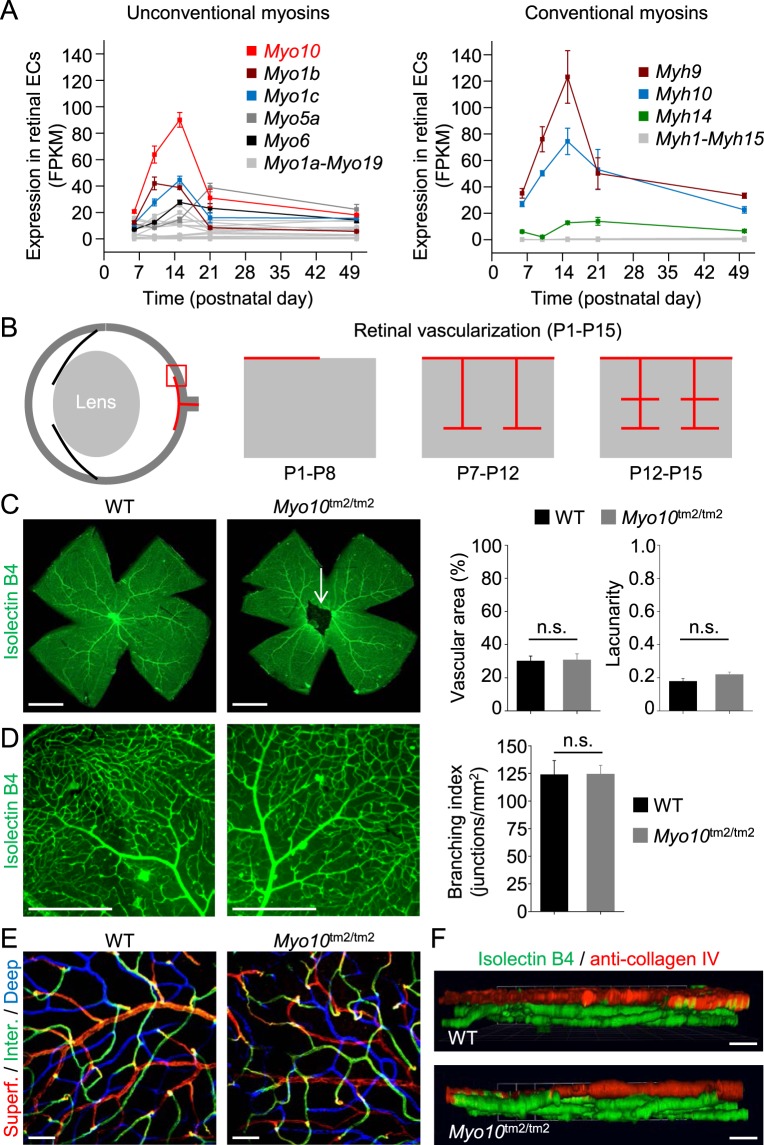
*Myo10* expression in retinal vascular endothelial cells and retinal vascularization in *Myo10*^tm2/tm2^ mice. (**A**) Expression profile of the myosin superfamily in postnatal retinal endothelial cells. Notably, RNA sequence analysis revealed increased expression of *Myo10* between postnatal day 6 (P6) and P15 (n = 3 independent preparations per group). (**B**) Schematic diagram showing vascularization of the deeper layers of the retina between P7 and P15. (**C**) Whole-mount retinas from wild-type (WT) and *Myo10*^tm2/tm2^ mice stained for blood vessels (representative of 6 preparations for each genotype). The white arrow indicates persistent hyaloid vessels. Quantitative analysis of the vascularization of WT versus *Myo10*^tm2/tm2^ retinas is shown on the right (n = 6 retinal preparations (from 3 mice) for each group). Vascular area is the percentage of area covered by the vascular network and lacunarity is a measure of heterogeneity. Scale bars: 1 mm. (**D**) Higher magnification images of isolectin B4-stained retinal blood vessels. Quantitative analysis of vessel branching (branch points/unit area) is shown on the right. Scale bars: 500 µm. (**E**) Higher magnification images (obtained using a 20x/0.45 objective lens) of retinal vascular layers, color coded blue (deep plexus), green (intermediate (inter.) plexus) and red (superficial (superf.) plexus). Scale bars: 50 µm. (**F**) Side-view projections of 3D reconstructions of retinal vessels, obtained by spinning disk confocal microscopy (via a 20x/0.45 objective lens). Anti-collagen, type IV, stained the superficial plexus (red), whereas all vessels were labeled with isolectin B4 (green). Scale bars: 100 µm.

In addition to persistence of the hyaloid vasculature, mice lacking the Norrie disease protein (Ndp) Norrin, encoded by *Ndp*, develop retinal hypovascularization, which is phenocopied in mice lacking *Fzd4* or *Lrp5* (Fig. 8C). We therefore investigated whether mice lacking full-length Myo10 develop retinal hypovascularization in addition to persistence of the hyaloid vessels (Fig. 9). RNA sequence analysis (next generation sequencing) of ribosome-bound RNA isolated from postnatal retinal endothelial cells at P6, P10, P15, P21 and P50, respectively, revealed that *Myo10* (full-length isoform) is the most abundantly expressed unconventional myosin (Fig. 9A). Between P6 and P15, *Myo10* expression increased more than 4-fold (Fig. 9A), when vertical sprouting angiogenesis drives the formation of a deep vascular plexus (P7-P12), followed by intermediate vascular plexus formation (P12-P15), as schematically illustrated in Fig. 9B. We speculated that Myo10 may be important for the formation of filopodia at tip cells, the cells at the tips of vascular sprouts^45^. However, imaging of whole-mount retinas by fluorescence stereomicroscopy and spinning disk confocal microscopy did not reveal impaired vascularization in adult retinas (Fig. 9C,D). Deeper vascular layers could be clearly detected in retinas from *Myo10*^tm2/tm2^ mice (Fig. 9E,F). Thus, *Myo10*^tm2/tm2^ mice show persistence of the hyaloid vasculature without retinal hypovascularization.

### Macrophages from *Myo10*^tm2/tm2^ mice do not have impaired Fcγ receptor-mediated phagocytosis

**Figure 10:**
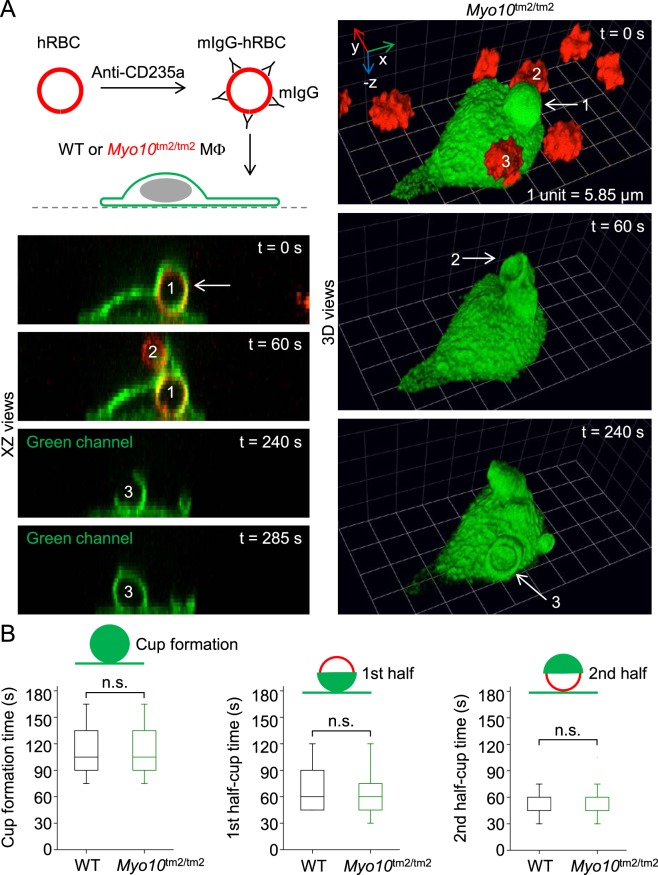
Myo10 is not important for Fcγ receptor-mediated phagocytosis. (**A**) Model system for Fcγ receptor-mediated phagocytosis. Time-lapse images (XZ and reconstructed 3D views), obtained by spinning disk confocal microscopy, of a *Myo10* knockout (*Myo10*^tm2/tm2^) macrophage engulfing freshly isolated hRBCs (human red blood cells) opsonized with mouse immunoglobulin G (mIgG). At t = 0 s, a mIgG-hRBC has already been ingested (labeled 1). At t = 60 s, a nascent phagocytic cup, extending from the first cup, is forming around a second hRBC (labeled 2). A third cup, emerging from the cell body, can be seen at t = 240 s. (**B**) Kinetics of phagocytic cup formation. XY, XZ and YZ views of the confocal data sets (n = 19–38 events per group; 2 independent experiments) were used to measure the kinetics of single phagocytic cup formations.

We recently reported that resident peritoneal macrophages from *Myo10*^tm2/tm2^ mice generate less nascent filopodia compared to wild-type cells, but have no defects in the phagocytosis of zymosan or immunoglobulin G (IgG)-coated polystyrene beads^46^. This was surprising since Myo10 had been previously implicated in Fcγ receptor-mediated phagocytosis^26^. Since macrophages can ingest unopsonized polystyrene beads, used in our previous study, we re-investigated Fcγ receptor-mediated phagocytosis using an alternative assay in which macrophages are presented with freshly isolated human red blood cells (hRBCs) coated (opsonized) with mouse anti-CD235a antibodies (mIgG; illustrated in Fig. 10A). Notably, we confirmed that mouse peritoneal macrophages do not ingest unopsonized hRBCs. In assays using macrophages isolated from NOTAM mice, which harbor a γ-chain mutation^47^, or *Fcer1g*-deficient mice, we have confirmed that mIgG-hRBCs are engulfed exclusively via Fcγ receptors (unpublished data). Using time-lapse spinning disk confocal microscopy, we found that macrophages isolated from *Myo10*^tm2/tm2^ mice had no defects in phagocytic cup formation or the ingestion of mIgG-hRBCs (Fig. 10A,B). Thus, Fcγ receptor-mediated phagocytosis is not impaired in macrophages lacking full-length Myo10.

**Figure 11:**
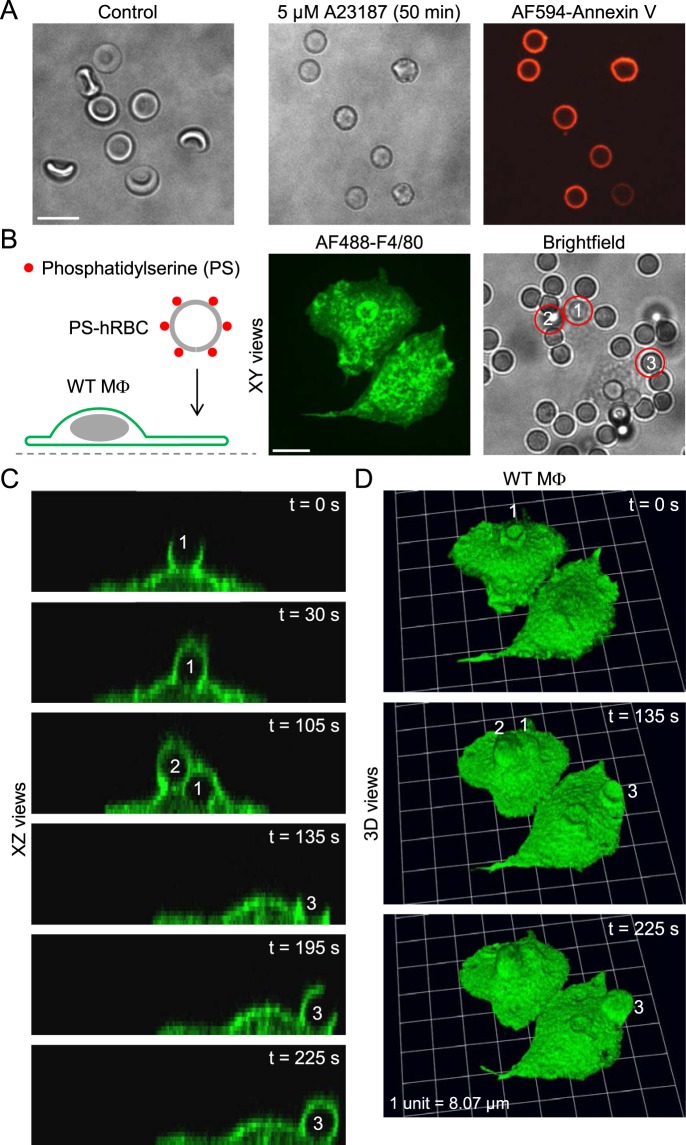
Assay to image the phagocytosis of apoptotic human red blood cells. (**A**) Brightfield images of control and A23187-treated human red blood cells, and labeling of phosphatidylserine with Alexa Fluor 594-conjugated annexin V (AF594-Annexin V; red channel). Scale bar: 10 µm. (**B**) Introduction of human red blood cells (hRBCs) with externalized phosphatidylserine (PS) to wild-type (WT) mouse peritoneal macrophages (MΦs). Scale bar: 10 µm. (**C** and **D**) Time-lapse images (XZ and reconstructed 3D views) of macrophages ingesting the three hRBCs (labeled 1,2 and 3, respectively) shown in the brightfield image of panel B.

**Figure 12:**
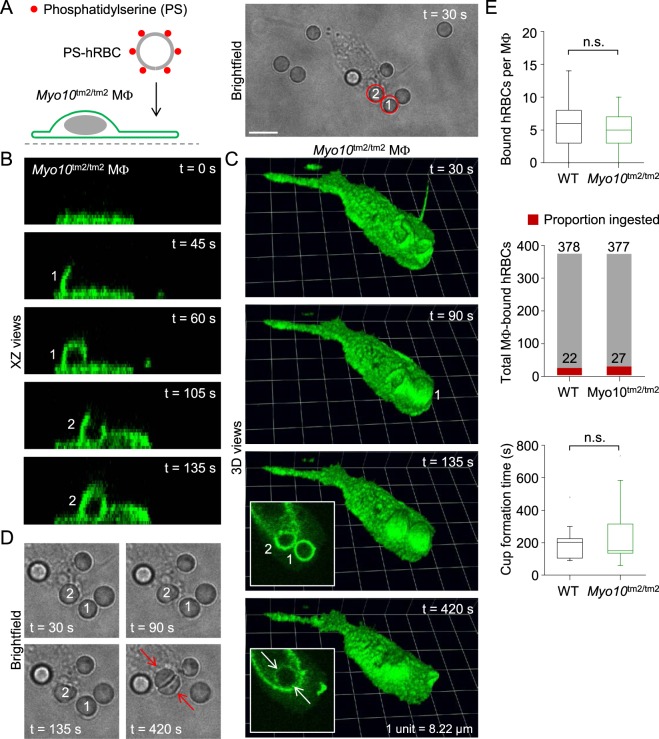
Ingestion of apoptotic human red blood cells by a *Myo10*^tm2/tm2^ macrophage. (**A**) Introduction of human red blood cells (hRBCs) with externalized phosphatidylserine (PS) to a *Myo10*^tm2/tm2^ peritoneal macrophage (MΦ). Scale bar: 10 µm. (**B** and **C**) Time-lapse images (XZ and reconstructed 3D views) of a *Myo10*^tm2/tm2^ macrophage ingesting the two hRBCs (labeled 1 and 2, respectively) shown in the brightfield image of panel A. (**D**) Time-lapse brightfield images showing two human red blood cells being packed together after their respective phagosomes, shown in the insets of panel C, have fused. (**E**) Summary data for wild-type (WT; n = 3) versus Myo10^tm2/tm2^ (n = 2) mice. n.s. = not significant.

Next, we tested whether Myo10 is important for the engulfment of apoptotic cells. We induced externalization of phosphatidylserine, an “eat me” signal, in human red blood cells by incubation with 5 µM A23187, a Ca^2+^ ionophore, for 50 min at 37 °C (Fig. 11A). After a wash step, the red blood cells were resuspended in annexin-binding buffer (Fig. 11A). This protocol consistently led to phosphatidylserine externalization, confirmed by incubating cells with Alexa Fluor 594-conjugated annexin V (Fig. 11A). We did not use N-ethylmaleimide to enhance phosphatidylserine externalization, as described by Closse *et al*.^48^, since we found that this compound is highly toxic to mouse macrophages. Human red blood cells with externalized phosphatidylserine adhered to macrophages, but only a small subset of particles were engulfed (Fig. 11B–D). This suggests that exposure of the phospholipid phosphatidylserine alone is probably not sufficient to trigger phagocytosis, a controversial issue^49^. Similar to wild-type cells, *Myo10*^tm2/tm2^ macrophages sporadically ingested human red blood cells with phosphatidylserine exposure (Fig. 12A–D). The example in Fig. 12 nicely shows how the phagosomes of two ingested human red blood cells fuse (insets in Fig. 12C), such that the two blood cells become pressed together (Fig. 12D). There were no significant differences, wild-type versus *Myo10*^tm2/tm2^ macrophages, in the number of adherent human red blood cells per macrophage and the rates of phagocytic cup formation, although phagocytic events were infrequent in both groups (Fig. 12E).

## Discussion

We investigated the *in vivo* function of the MyTH4-FERM myosin Myo10 using *Myo10* reporter knockout mice. The *Myo10* reporter knockout allele (*Myo10*^tm2^) lacks exon 19 and harbors a gene trap. We confirmed that full-length (motorized) Myo10 was deleted in homozygous mutant (*Myo10*^tm2/tm2^) mice, but the headless isoform of Myo10 was still expressed in the brain, even though one (namely, NM_001353142.1) of the two confirmed Hdl-Myo10 transcripts is expected to be marginally truncated at the 5′-UTR in the *Myo10*^tm2^ allele (Fig. 1D). Thus, *Myo10*^tm2/tm2^ mice are not simply *Myo10* null mice, but selectively lack the full-length, motorized isoform. *Myo10*^tm2/tm2^ mice exhibited four phenotypes (percent penetrance indicated): (1) exencephalus (24%), a cranial neural tube closure defect, (2) pigmentation defects (white belly spots; >95%), (3) simple syndactyly (>95%), and (4) persistence of the hyaloid vasculature (>95%). As alluded to in the Introduction, the phenotypes of both Myo10 null (Myo10^tm1d/tm1d^) mice^27^ and Myo10^tm2/tm2^ mice^28^ were recently reported. These studies nicely confirmed that Myo10 is important for neural tube closure^27^, and that Myo10 deficiency leads to pigmentation defects, syndactyly and persistence of the hyaloid vasculature^27,28^. We discuss each of these phenotypes below, as well as putative roles of Myo10 in other contexts.

### Exencephalus

Neurulation, the folding of the neural plate to form a neural tube, involves expansion of the mesoderm and elevation of the neural folds, followed by dorsolateral bending^50^. At E8.5, the folds close at the hindbrain-cervical boundary (closure 1) and zippering spreads rostrally and caudally. A second closure (closure 2) at the forebrain-midbrain boundary initiates a second site of zippering. Failure or disruption of closure 2-mediated zippering leads to exencephalus^50^. We speculate that susceptibility to exencephalus in *Myo10*^tm2/tm2^ embryos may be due to impaired adhesion of opposing neural fold apices since cell-cell adhesion molecules implicated in tube closure, including cadherins, are known cargo proteins for Myo10^12,13,51^. Moreover, numerous filopodia, the hallmark of Myo10, have been shown to extend from the non-neural ectoderm and bridge opposing neural folds^52^; see also review by Nikolopoulou *et al*.^53^. Genetic experiments in mice have strongly implicated N-cadherin (neural cadherin) in neural tube closure. N-cadherin null embryos rescued by cardiac N-cadherin expression, as well as neural crest-restricted N-cadherin knockout embryos, exhibit exencephalus due to failed closure of the anterior neuropore^54,55^. In this context, it would be interesting to test whether neural crest-restricted deletion of *Myo10* predisposes to, firstly, exencephalus, due to loss of N-cadherin transport, and, secondly, pigmentation defects, due to decreased generation or migration of neural crest-derived melanoblasts. Interestingly, the incidence of exencephalus is higher (68% versus 24%) in *Myo10* null (*Myo10*^tm1d/tm1d^)^27^ versus *Myo10*^tm2/tm2^ mice^28^. Thus, headless Myo10, which strongly localizes to the plasma membrane (see Figs 3 and 4), may partially compensate for loss of full-length Myo10 during neurulation. X-gal staining for *Myo10* expression in *Myo10*^tm2^ embryos appeared negative at the neural fold (see Fig. 2E). However, the expression of headless-*Myo10* is probably not reported in *Myo10*^tm2^ mutants since the *lacZ* reporter gene is located 5′ upstream of headless *Myo10* transcripts (see Fig. 1), whereas both full-length and headless *Myo10* are expected to be reported by X-gal staining in *Myo10*^tm1a^ embryos^27^.

### Pigmentation defects (white belly spots)

During neural tube closure, neural crest cells delaminate from the apices of the neural folds and undergo epithelial-to-mesenchymal transition. Neural crest cells are highly migratory and give rise to diverse cell lineages, including melanocytes (pigment-producing cells), under the control of a network of regulatory transcription factors and downstream effector genes^56^. Neural crest-derived melanoblasts, the precursors of melanocytes, migrate dorsolaterally, populate the ectoderm and subsequently colonize hair follicles, where the cells produce the pigment melanin. *Myo10*^tm2/tm2^ mice consistently showed white belly spots, and histology confirmed that the spots are devoid of melanocytes (see Fig. 6). *Myo10* is expressed in neural crest cells^57^ and has been reported to be a signature gene of epidermal neural crest stem cells^58^. However, aside from white belly spots, *Myo10*^tm2/tm2^ mice did not exhibit major neural crest-related disorders, such as craniofacial defects or megacolon (intestinal aganglionosis)^59^. We speculate that *Myo10* may be an effector gene for the transcription factors specifying the melanocyte lineage, such as Sox10, Pax3 and Mitf, and may confer motility. In *Xenopus laevis*, Myo10 is a neural crest signature gene^60^ and knockdown of the gene has been reported to decrease neural crest cell migration^24,25^. Furthermore, melanoblast-restricted deletion of the filopodia-inducing Rho GTPase Cdc42 impairs melanoblast motility and mice develop severe belly pigmentation defects, similar to Rac1 conditional knockout mice^61,62^. It would be useful to test whether neural crest- (as alluded to above) or melanoblast-restricted deletion of *Myo10* phenocopies homozygous *Myo10*^tm2^ mutants. The presence of white belly spots was a predictor of genotype (>95% of *Myo10*^tm2/tm2^ mice had white belly spots). This high frequency agrees with the 100% penetrance reported by Heimsath *et al*.^27^. (*Myo10*^tm1d/tm1d^ mice) and Tokuo *et al*.^28^ (*Myo10*^tm2/tm2^ mice), as well as the high rate reported by Liakath-Ali *et al*. for *Myo10*^tm2/tm2^ mice^63^.

### Syndactyly

During embryonic development in the mouse, digit formation involves the initiation and progression of interdigital cell death. BMPs (bone morphogenetic proteins), secreted proteins, are thought to play an important role in initiating interdigital cell death, which involves inhibition of anti-apoptotic fibroblast growth factors and Wnt signaling. Consistent with this scheme, conditional deletion of *Bmp2* (bone morphogenetic protein 2) in limb bud mesenchyme in mouse leads to soft tissue syndactyly between digits 3 and 4, with variable penetrance, whereas conditional deletion of both *Bmp2* and *Bmp4* produces complete syndactyly of fore- and hindlimbs^64^. Mice lacking *Dkk1* (dickkopf Wnt signaling pathway inhibitor 1) or *Sfrp2* (secreted frizzled-related protein 2), genes encoding soluble inhibitors of Wnt signaling, develop syndactyly. In *Sfrp2*−/− mice, digits 3 and 4 of the hindlimb are consistently fused^65^, whereas digits 2 and 3 are fused in *Dkk1*−/− mice^66^, among other defects. Similarly, digits 2 and 3 or 3 and 4 are fused in *Myo10*^tm2/tm2^ mice with high penetrance (~95%); in contrast, Tokuo *et al*.^28^ reported 72% penetrance for *Myo10*^tm2/tm2^ mice, and Heimsath *et al*.^27^ reported ~50% for *Myo10*^tm1d/tm1d^ mice. A different pattern of syndactyly is seen when the intrinsic pathway of apoptosis, mediated by cytochrome *c* release and caspase 9 activation, is inhibited in mice. Instead of fusions affecting digit pairs 2 and 3 or 3 and 4, persistent interdigital webbing is observed in mice lacking both (double knockout) Bax (encoded by *Bax*) and Bak (*Bak1*)^67^, required for cytochrome *c* release, or mice lacking the three (triple knockout) Bax/Bak activators Bid (*Bid*), Bim (*Bcl2l11*) and Puma (*Bbc3*)^68^. Thus, Myo10 probably regulates the regression of interdigital mesenchyme, presumably as a downstream effector of Bmp signaling^21^, by initiating interstitial cell death. Impaired phagocytic clearance in *Myo10*^tm2/tm2^ mice is unlikely to explain the syndactyly phenotype, since we found that *Myo10*^tm2/tm2^ macrophages have robust phagocytic cup formation and rapidly ingest large IgG-coated beads^46^ or IgG-coated red blood cells, as well as apoptotic cells (see Figs 10 and 12). Moreover, impaired phagocytosis would probably give rise to persistent webbing rather than complete or near complete syndactyly of selected digits. Interestingly, syndactyly is not observed in embryos of PU.1 null mice, which lack macrophages, since mesenchyme cells can assume the function of phagocytes in the interdigital space^69^. However, the hyaloid vasculature fails to regress in PU.1 null mice^41^.

### Persistence of the hyaloid vasculature

*Myo10*^tm2/tm2^ mice consistently exhibited persistence of hyaloid vessels, as reported for *Myo10*^tm1d/tm1d^ mice^27^. The hyaloid vasculature normally regresses before eye opening, which occurs around P10 - P13. As alluded to in the Results section, regression of the hyaloid vessels requires stimulation of Fzd4 and its co-receptor Lrp5, expressed on vascular endothelial cells, by the ligands Wnt7b and Norrin. Macrophages have been deduced to be the source of Wnt7b as well as the initiators of pro-apoptotic canonical Wnt signaling in hyaloid vascular endothelial cells^41^. Mice lacking Wnt7b, Norrin, Fzd4 or Lrp5 have hyaloid vessel persistence^41–43,70^. Mice lacking Norrin, Fzd4 or Lrp5 additionally show retinal hypovascularization^42,71,72^, which has not been reported for mice homozygous for the hypomorphic allele Wnt7b^d1^ ^41^. We anticipated that *Myo10*^tm2/tm2^ mice would exhibit retinal hypovascularization since we found that full-length *Myo10* expression increased more than 4-fold in retinal vascular endothelial cells between P6 and P15 (see Fig. 9A,B), when sprouting angiogenesis forms deep and intermediate vascular plexuses. However, we could not detect impaired retinal vascularization in whole-mount retinal preparations from adult *Myo10*^tm2/tm2^ mice. Thus, hyaloid vessel persistence cannot be explained as a secondary consequence of insufficient retinal vascularization^73^. How Myo10 regulates hyaloid regression is unclear, although we found that full-length Myo10 is expressed in both macrophages^46^, which trigger hyaloid regression^41^, and in retinal vascular endothelial cells. Interestingly, long filopodia and Myo10-mediated transport have been implicated in Wnt signaling^74^, implying that Myo10 may be involved in the cell death-inducing Wnt signaling between retinal microglia (macrophages) and hyaloid vessels^41^. Imaging of the pupillary membrane, a transient structure on the anterior surface of the lens, in postnatal mice has impressively shown that numerous long, thin filopodia extend from vascular endothelial cells and interact with resident macrophages^75^. However, whether Myo10-induced filopodia formation is important for endothelial cell-macrophage communication and programmed capillary regression remains to be clarified. In HEK293T cells, we found that Fzd4-EGFP localized to the plasma membrane, but, in contrast to DCC, it was not enriched at the tips of Myo10-induced filopodia (not shown).

### Axon guidance

Myo10 has been implicated in neuritogenesis and axon guidance^23,30,76^, and mice lacking the Myo10 cargo protein DCC fail to form commissures in the brain^34^. However, MRI of isolated, fixed brains revealed that the corpus callosum, anterior commissure and hippocampal commissure were intact in *Myo10*^tm2/tm2^ mice (see Fig. 5C). These observations suggest that full-length Myo10 is redundant for Netrin-1-mediated axon pathfinding. We confirmed that headless Myo10, which is still expressed in *Myo10*^tm2/tm2^ mice, does not induce filopodia, but we found that headless Myo10 robustly localizes to the plasma membrane independent of the MyTH4-FERM domain. This was somewhat surprising since GFP-tagged headless Myo10 has been reported to more diffusely localize in CAD (Cath.a-differentiated) cells^29^, a neuronal cell line. Thus, in addition to dimerization with full-length Myo10 and competition for cargo^29^, headless Myo10 may negatively regulate full-length Myo10 by masking membrane phosphoinositides.

## Summary and Conclusions

We found that the expression of full-length Myo10, but not headless Myo10, is abolished in Myo10^tm2/tm2^ mice. Homozygous mutant embryos developed exencephalus, a lethal phenotype, with low penetrance, whereas surviving *Myo10*^tm2/tm2^ mice consistently exhibited white belly spots, simple syndactyly and persistence of the hyaloid vasculature without retinal hypovascularization. We confirmed *in vitro* that DCC localizes with Myo10 at the tips of filopodia, but *in vivo* we could not detect defects in the brain commissures of *Myo10*^tm2/tm2^ mice, in contrast to *Dcc* knockout mice. We also showed *in vitro* that headless Myo10 strongly localizes to the plasma membrane in a PH domain-dependent fashion. The unusual combination of pigmentation defects and syndactyly observed in *Myo10*^tm2/tm2^ mice is reminiscent of variants of Waardenburg syndrome, and we suspect that *Myo10*^tm2/tm2^ mice develop progressive hearing loss. The phenotypes of hyaloid persistence and syndactyly suggest that Myo10 may be important for initiating apoptosis, which is also required for neural tube closure^77^. Intact phagocytosis by *Myo10*^tm2/tm2^ macrophages *in vitro* suggests that phagocytic clearance is not impaired in homozygous mutant mice.

## Materials and Methods

### Mice

Heterozygous *Myo10* reporter knockout (*Myo10*^+/tm2^) mice were generated by the Wellcome Trust Sanger Institute (Wellcome Trust Genome Campus, Hinxton, Cambridgeshire, CB10 1SA, United Kingdom), as part of the KOMP (Knockout Mouse Project). The targeted allele is also denoted *Myo10*^tm2(KOMP)Wtsi^, where Wtsi is Wellcome Trust Sanger Institute. The targeting sequence replaces exon 19 of *Myo10* and contains a *lacZ* reporter and gene trap (polyadenylation (pA) site), as well as a neomycin resistance cassette flanked by loxP sites. No experiments were performed on live vertebrates. All methods were carried out in accordance with the relevant guidelines and regulations, and all experimental protocols were approved by the Landesamt für Natur, Umwelt und Verbraucherschutz Nordrhein-Westfalen, Germany.

### Southern blot analysis

Mouse tail biopsies were lysed overnight at 55 °C in buffer containing 100 mM Tris-HCl (pH 8.5), 5 mM EDTA, 0.2% sodium dodecyl sulfate, 200 mM NaCl, and 100 μg/ml proteinase K. After phenol/chloroform extraction, DNA samples were precipitated by isopropanol, washed in 80% ethanol, dried and dissolved in 50 μl TE Buffer (10 mM Tris (pH 7.9) and 0.2 mM EDTA). Approximately 5 μg genomic DNA was digested with *Bam*HI or *Eco*RI restriction endonuclease, fractionated on 0.8% agarose gels, and transferred to GeneScreen nylon membranes (NEN-DuPont, Boston, MA). The membranes were hybridized with a ^32^P-labeled 2.4 kb probe containing sequences 5′ to the targeted homology and washed with (final concentrations) 0.5x SSPE (1x SSPE contains 0.18 M NaCl, 10 mM NaH_2_PO_4_, and 1 mM EDTA; pH 7.7) and 0.5% sodium dodecyl sulfate at 65 °C. The hybridization probe was cloned as follows. A DNA PCR product was amplified from mouse genomic DNA using the oligonucleotide pair Myo10HR1d and Myo10HR1r and cloned into a custom vector using *Bsm*BI restriction endonuclease sites, followed by sequencing for verification. The sequence of the Myo10HR1d oligonucleotide was 5′-*GCTCTAGACGTCTCTGAGATGAGATGATCA*GGTCCTGGTGTTA-3′, and Myo10HR1r was 5′-*GTCTCAAGCGTCTCTTGGA*CATTCTAATATCCTGTATACCCCTCACA-3′. The sequences used for cloning of the PCR product are in italics.

### Genotyping

PCR for genotyping the *Myo10* reporter knockout mice was performed in two steps. First, touchdown PCR was performed using the following thermocycling protocol: 94 °C for 5 min, then 6 cycles of 94 °C for 30 s, 61 °C (with subtraction of 1 °C per cycle) for 30 s, and 72 °C for 60 s. This was followed by 31 cycles of 94 °C for 30 s, 57.5 °C for 30 s, and 72 °C for 60 s. The final extension was 72 °C for 5 min, followed by a holding temperature of 12 °C. The following primer sequences (5′ → 3′) were used: Myo10_F, ATCTGTTTCCCCTTAAGCGAAAAT; Myo10_R, CTCTGTGGGGCCCAGAGCT; CAS_R1_Term, TCGTGGTATCGTTATGCGCC. The expected band (product) size for the primer pair Myo10_F and CAS_R1_Term was 295 bp (mutant allele), and the expected size for the pair Myo10_F and Myo10_R was 400 bp (wild-type allele). *LacZ* could also be detected using the primer pair LacZ_2_small_F (ATCACGACGCGCTGTATC) and LacZ_2_small_R (ACATCGGGCAAATAATATCG), which had an expected band size of 108 bp (*LacZ* positive).

### Western blot analyses

Myo10 protein expression was analyzed using Western blot (protein immunoblot). Mouse P10 brain tissue was homogenized in buffer (1 ml per 100 mg tissue) containing: 15 mM Hepes (pH 7.4) and 320 mM sucrose, supplemented with leupeptin (protease inhibitor), Pefabloc SC (proteinase K inhibitor) and aprotinin (inhibitor of trypsin and related proteases). The homogenate was centrifuged at 10000 x g for 10 min at 4 °C. The supernatant was aspirated, mixed 1:4 with 5x Laemmli buffer and heated at 95–99 °C for 5 min. The Laemmli (Lämmli) buffer contained SDS (sodium dodecyl sulfate), β-mercaptoethanol, and the tracking dye bromophenol blue. HEK293T cells overexpressing Myo10 constructs were lysed using Cell Lysis Buffer (#9803; Cell Signaling Technology, Leiden, The Netherlands) containing: 20 mM Tris-HCl (pH 7.5), 150 mM NaCl, 1 mM Na_2_EDTA, 1 mM EGTA, 1% Triton, 2.5 mM sodium pyrophosphate, 1 mM β-glycerolphosphate, 1 mM Na_3_VO_4_ (phosphatase inhibitor) and 1 μg/ml leupeptin, supplemented with 5 mM NaF (phosphatase inhibitor), 10 μg/ml aprotinin, 10 μg/ml Pefabloc SC and 1 mM DTT (dithiothreitol). As in the case of tissue lysates, cell lysates were mixed with Laemmli buffer and heated.

Proteins were separated in gels of 6.5% polyacrylamide by electrophoresis and subsequently transferred overnight by tank blot onto polyvinylidene difluoride membranes (Roche, Mannheim, Germany). After blocking with 5% milk powder in TBST (Tris-buffered saline with Tween 20, containing 50 mM Tris, 150 mM NaCl and 0.1% Tween 20; pH 8.0) for 1 h at room temperature, primary antibody (mouse monoclonal anti-Myo10 antibody; sc-166720, Santa Cruz Biotechnology) was added for 24–48 h at 4 °C. Secondary antibody, horseradish peroxidase-conjugated anti-mouse antibodies, was added for 1 h at room temperature after 3 × 15 min washes with TBST. Finally, SuperSignal West Pico chemiluminescence substrate (Perbio, Bonn, Germany) was applied and immunoblot images were captured using a ChemiDoc MP imaging system (Bio-Rad, Bio-Lab Laboratories, München, Germany).

### Plasmids and cloning

A mammalian expression vector (pCMV-Tag2B) harboring N-terminal FLAG-tagged, full-length mouse Myo10 (pCMV-Tag2B-mMyo10) was kindly provided by Thomas B. Friedman (Bethesda, MD). A headless Myo10 (Hdl-mMyo10) construct in the same vector (pCMV-Tag2B-Hdl-mMyo10) was generated by combining DNA fragments from PCR (using the forward primer (with EcoRI overhang) 5′-AGTCCGAATTCATGACAGACCAGTTTGATCAGGTG-3′ and the reverse primer 5′-TGTAAACCCTGCGTGCCAGC-3′) and digestion with restriction enzymes (EcoRI and SalI). Hdl-mMyo10 was subcloned from the parent vector pCMV-Tag2B-mMyo10 into the vector pEGFP-C1 (Clontech Laboratories), which fuses EGFP to the N-terminus, using the same approach, except the forward primer (with BglII overhang) was 5′-ACTGAGATCTATGCCAGACCAGTTTGATCAGGTG-3′ and the plasmid pCMV-Tag2B-mMyo10 was digested with BstEII (also known as Eco91l) and SalI. The following Hdl-mMyo10 deletion constructs were generated using the template pEGFP-C1-Hdl-mMyo10: EGFP-Hdl-mMyo10ΔMyTH-FERM, EGFP-Hdl-mMyo10ΔPH-MyTH-FERM and EGFP-Hdl-mMyo10ΔPH3-MyTH-FERM. pEGFP-mMyo10 (full-length mouse Myo10) was also derived from pCMV-Tag2B-mMyo10.

Expression vectors for human Myo10 with EGFP fused at the C-terminus (pEGFPN3-hMyoX (hMyo10-EGFP); plasmid #47609, deposited by Emanuel Strehler), human DCC (pCMV-DCC; plasmid #16459, deposited by Bert Vogelstein), and mouse Frizzled 4 with EGFP fused at the C-terminus (pFz4-GFP (Fzd4-EGFP); plasmid #42197, depositied by Robert Lefkowitz and Jeremy Nathans) were obtained from the plasmid repository Addgene. The vector for expression of human Wnt7b tagged with tGFP (TurboGFP) at the C-terminus (pCMV6-AC-Wnt7b-tGFP) was obtained from OriGene (MG205288; Herford, Germany). The plasmid for expression of bovine Myo10 (bMyo10) tagged with mCherry at the N-terminus (pmCherry-bMyo10) was kindly supplied by Staffan Strömblad (Huddinge, Sweden) and has been previously described by Plantard *et al*.^78^.

### Transfection and imaging

HEK293T cells were plated on human fibronectin-coated 35 mm glass (or polymer) bottom cell culture dishes (Ibidi, Martinsried, Germany) and incubated in DMEM (Dulbecco’s Modified Eagle’s Medium) supplemented with 10% bovine serum albumin and penicillin/streptomycin. Cells were transfected with calcium phosphate (Myo10 and DCC (deleted in colorectal cancer) constructs), PolyFect transfection reagent (Qiagen) or Lipofectamine LTX (Thermo Fisher Scientific). DCC was labeled with mouse anti-DCC (monoclonal) antibodies which recognize the extracellular domain of human DCC protein (ab16793; Abcam, Cambridge, UK). The plasma membrane was stained by incubating cells with CellMask Orange (diluted 1:1000) for 5 min at 37 °C. Live-cell imaging was performed, via a Nikon Apochromat TIRF 60x/1.49 (oil-immersion) objective lens, using a spinning disk confocal microscope (UltraVIEW Vox 3D live cell imaging system coupled to a Nikon Eclipse Ti inverse microscope; Perkin Elmer, Rodgau, Germany). The system included a Yokogawa (Japan) CSU-X1 spinning disk scanner, a Hamamatsu (Japan) C9100-50 EM-CCD camera (1000 × 1000 pixels) and Volocity software.

### Superresolution structured illumination microscopy

Superresolution structured illumination microscopy was performed using an Elyra S.1 inverted microscope system (Carl Zeiss MicroImaging, Germany), controlled by ZEN 2011 SP2 software (black edition; Zeiss). Cells were imaged via a Plan Apo 63/1.4 (oil-immersion) objective lens and images were captured with an Andor iXon EM-CCD. The following lasers and filters (in parentheses) were used: 488 nm (BP 495–550 nm + LP 750 nm) and 561 nm (BP 570–620 + LP 750 nm). Five grating positions and 5 phase shifts were used for each z-slice. Note that cells fixed after transfection were counterstained with Alexa Fluor 594-conjugated phalloidin (Invitrogen) to allow imaging of F-actin.

### X-gal staining

Whole-mount mouse embryo X-gal (5-bromo-4-chloro-3-indolyl-β-D-galactopy-ranoside) staining was performed using a LacZ tissue staining kit (rep-lz-t; InvivoGen). X-gal is a chromogenic substrate for β-galactosidase, encoded by the *LacZ* gene. Embryos were fixed for 30–60 min (E10.5–14.5) at room temperature using the following fixative solution: 1% neutral buffered formalin, 0.2% glutaraldehyde, 2 mM MgCl_2_, 5 mM EDTA and 0.02% IGEPAL CA-630, a nonionic, non-denaturing detergent, in PBS. After several wash steps using 0.02% IGEPAL CA-630 in PBS (pH 8.5), fixed embryos were stained in the dark for 30–120 min at 37 °C (or room temperature when staining digits) with staining solution: 5 mM K_3_Fe(CN)_6_, 5 mM K_4_Fe(CN)_6_, 2 mM MgCl_2_, 0.02% IGEPAL CA-630 and 1 mg/ml X-gal in PBS (pH 8.5). After staining, embryos were washed 2 × 10 min in PBS containing 0.1% Tween-20 at room temperature, followed by 18 h incubation in the dark with post-fixative (10% neutral buffered formalin). When required, formalin-fixed, X-gal-stained embryos were embedded in paraffin wax and sectioned with a microtome, followed by deparaffination and rehydration. Sections were counterstained with the pink stain eosin.

### Magnetic resonance imaging

MRI measurements of *ex vivo* samples were performed on a 9.4 T BioSpec 94/20 system (Bruker BioSpin, Ettlingen, Germany). Tissue samples were fixed in 4% paraformaldehyde (PFA) in PBS for at least 24 h. Prior to MRI, samples were equilibrated in PBS containing 2 mM Gd-DTPA (Magnevist, Bayer Schering Pharma AG, Germany) and finally embedded in 1% low-melting agarose containing 2 mM Gd-DTPA.

Fixed mouse eyes were measured in 1 mL PCR tubes, using a 0.7 T/m gradient system and a two element cryogenic surface coil (Bruker). The inner structures of the eye were visualized using a three-dimensional (3D) fast low-angle shot sequence in a total scan time of 5:23 hours with the following parameters: isotropic resolution, 23 µm; matrix size, 240 × 256 × 256; field of view, 5.5 × 6.0 × 6.0 mm; TR/TE, 37/4.5 ms; flip angle, 10 degrees; and averages, 8.

Fixed brains were imaged using a 1 T/m gradient system and a quadrature volume coil with an inner diameter of 35 mm (Rapid Biomedical, Rimpar, Germany). A 3D spin echo data set was acquired in 16:31 hours, using a turbo RARE sequence: isotropic resolution, 55 µm; matrix size, 296 × 232 × 232; field of view, 16 × 12.8 × 12.8 mm; TR/TE, 750/40 ms; RARE factor 12; and averages, 18.

### Histology

Harvested skin samples were placed (dermis side down) on Whatman filter paper, which preserves skin flatness during fixation, and then cut into strips. The strips were fixed in 10% neutral buffered formalin (Sigma-Aldrich), equivalent to about 4% formaldehyde, on ice. The next day, the tissue was dehydrated, embedded in paraffin wax and sectioned with a microtome. The formalin-fixed, paraffin-embedded sections were subsequently deparaffinized using the solvent xylene and graded washes with xylene and ethanol. Next, the sections were rehydrated with graded concentrations of ethanol in water. To optimize staining, antigen retrieval was performed by placing sections in target retrieval solution (Dako, Agilent Technologies, Santa Clara, CA), a modified citrate buffer (pH 6.1), and heated for 20 min in a pressure cooker. After washing and blocking with serum for 1 h, sections were incubated with primary antibody, goat anti-DCT (dopachrome tautomerase; also known as tyrosine-related protein 2) antibodies (sc-10451; Santa Cruz Biotechnology), for 2 h at room temperature. The sections were then incubated with biotinylated secondary antibody for 1 h at room temperature, washed and subsequently incubated with Vectastain ABC reagent (Vector Laboratories) before the addition of peroxidase substrate (alkaline phosphatase substrate kit, Vector Laboratories). Finally, the dark blue stain hematoxylin, which labels nucleic acid and other structures, was applied.

### Micro-computed tomography (µCT)

Microtomography of *ex vivo* samples was performed using a SkyScan 1176 system (Bruker microCT, Kontich, Belgium). Tissue samples were fixed in 4% PFA in PBS for 24 h, followed by 3 × 30 min wash steps in 70% ethanol at room temperature. Subsequently, samples were stored in 70% ethanol at 4 °C. Images were acquired using the X-ray source voltage set at 40 kV (range, 20–90 kV) and the energy (transmission) was attenuated using a 0.2 mm aluminium filter. The exposure time of the X-ray detector, cooled digital X-ray CCD camera (pixel size, 8.52 µm), was set to 780 ms (1 × frame averaging) and scanning performed at 0.5° rotation steps. Volumetric reconstruction of data sets was processed using SkyScan NRecon software (v1.6.9.8; Bruker microCT). The following reconstruction parameters were used: smoothing = 1 (range, 1–10), ring artifact reduction = 14 (range, 1–20), and beam-hardening correction = 36 (range, 1–100).

### RNA sequence (RNA-Seq) analysis

Expression profiling of retinal endothelial cells at five different postnatal days (P6, P10, P15, P21, and P50) was performed as recently described^79^. Pdgfb-iCre/Rpl22^HA/HA^ transgenic mice, which express hemagglutinin (HA) tagged ribosomal protein L22 (Rpl22) under control of Cre recombinase specifically expressed in endothelial cells, were generated by crossing inducible endothelial cell-specific Cre (Pdgfb-iCre) mice with Rpl22^tm1.1Psam^ RiboTag knock-in mice. Ribosome-bound transcripts were immunoprecipitated from whole retina lysates using anti-HA antibodies coupled to magnetic beads, and gene expression was analyzed by RNA-Seq analysis after performing quality control for endothelial cell-specificity using quantitative RT-PCR.

### Whole-mount retinal staining

The retina was dissected and whole-mount immunostaining performed as previously described by Pitulescu *et al*.^80^. In brief, eyeballs were enucleated and placed in 2.0 ml Eppendorf microcentrifugation tubes containing 4% PFA in PBS, and incubated for 2 h at room temperature in a tube rotator. After fixation, the eyes were washed with PBS, placed in a Petri dish and dissected under a stereomicroscope. Spring scissors with 8 mm blades (15003-08; Fine Science Tools) were used to both make an initial cut and excise the cornea. Next, two Dumont #5 forceps were used to remove the sclera and underlying choroid. Susequently, the lens was removed and four radial incisions were made to divide the retina into quandrants. The dissected retinas were placed in 2.0 ml tubes containing blocking and permeabilization buffer (1% bovine serum albumin and 0.3% Triton X-100 in PBS) and incubated at 4 °C overnight in a tube rotator. After washing (2 × 5 min) in PBlec buffer (1 mM CaCl_2_, 1 mM MgCl_2_, 0.1 mM MnCl_2_ and 0.4% Triton X-100 in PBS), each retina was incubated overnight at 4 °C with 1:25 Biotinylated GSL I-B_4_ isolectin (*Griffonia Simplicifolia* Lectin I isolectin (GSL I) B4; B-1205, Vector Laboratories, Burlingame, CA) and 1:200 rabbit anti-mouse collagen IV antibodies (2150-1470; Bio-Rad, Oxford, United Kingdom) diluted in PBlec buffer. Note that GSL I-B_4_ isolectin is a marker for mouse endothelial cells, useful for labeling deeper vascular plexuses, and the extracellular matrix protein collagen IV is a component of the basal lamina of blood vessels. Alexa Fluor 488-conjugated streptavidin (S11223; Thermo Fisher Scientific, Darmstadt, Germany), diluted 1:100, and secondary antibody (Alexa Fluor 546-conjugated donkey anti-rabbit IgG (H + L)), diluted 1:500, were introduced for 1.5 h at room temperature after washing 1 × 15 min with washing buffer (blocking and permeabilization buffer diluted 1:1 with PBS) and 3 × 10 min with PBS. The washing steps were repeated to remove unbound secondary antibodies. Stained retinas were transferred onto standard (25 mm × 75 mm) glass microscope slides via a plastic transfer pipette. For each retinal preparation, excess medium was aspirated and a glass coverslip (24 mm × 32 mm) containing a hanging drop of Fluoromount-G mounting medium (SouthernBiotech, Birmingham, AL) was gently applied.

Whole-mount, stained retinas were imaged at low magnification (x2) using a Leica MZ16 F fluorescence stereomicroscope. Higher magnification images were obtained via Nikon Plan Fluor ELWD 20x/0.45 (dry) and Apochromat TIRF 60x/1.49 (oil-immersion) objective lenses of a spinning disk confocal microscope (UltraVIEW Vox 3D live cell imaging system). Quantitative analysis of retinal vasculature was performed using AngioTool^81^.

### Live-cell phagocytosis assays

Mouse resident peritoneal macrophages were isolated and seeded into fibronectin-coated Ibidi µ-Slide I chambers as previously described^46^. After overnight incubation of the mouse macrophages, freshly isolated hRBCs (human red blood cells) were incubated with CellMask Orange (C10045; Thermo Fisher Scientific) plasma membrane stain (1:1000 dilution and 5 min incubation at 37 °C) and washed twice with bicarbonate-free RPMI 1640 medium containing 20 mM Hepes. The hRBCs were subsequently opsonized with mouse IgG by incubation for at least 8 min at 37 °C with (mouse) anti-CD235a monoclonal IgG (IgG2b; clone HIR2) antibodies (MA1-20893; Thermo Fisher Scientific), diluted 1:400. The opsonized hRBCs were not washed to avoid agglutination and directly pipetted into a µ-Slide I chamber seeded with macrophages freshly labeled (20 min pre-incubation at 37 °C, followed by wash) with rat anti-mouse F4/80 antibodies conjugated to Alexa Fluor 488 (1:40 dilution; MF48020, Thermo Fisher Scientific).

Phagocytosis of IgG-opsonized (red fluorescent) hRBCs by (green fluorescent) mouse macrophages was imaged by time-lapse spinning disk confocal microscopy. Z-stacks (22 slices at 0.8 µm steps) for each channel (488 nm (green channel) and 561 nm (red channel) laser excitation, respectively) were obtained every 15 s for 16 min. Notably, the bicarbonate-free RPMI 1640 medium (containing 20 mM Hepes) was supplemented with 1 mM MPG (*N*-(2-mercaptopropionyl)glycine), a free radical scavenger, to reduce phototoxicity. Focal drift was circumvented using the Nikon Perfect Focus Sytem.

### Phagocytosis of apoptotic cells

To induce externalization of phosphatidylserine, an “eat me” signal, hRBCs were incubated with the Ca^2+^ ionophore A23187 (5 µM) for 50 min, followed by wash steps. The pellet was resuspended in annexin binding buffer (V13246; Thermo Fisher Scientific), which contained 10 mM Hepes, 140 mM NaCl and 2.5 mM (pH 7.4). Externalization of phosphatidylserine was detected using Alexa Fluor 594-conjugated annexin V (A13203; Thermo Fisher Scientific). Notably, annexin V binds to phosphatidylserine in the presence of Ca^2+^. In phagocytosis assays, A23187 treated hRBCs, without CellMask Orange or red-fluorescent annexin V staining, were introduced to macrophages labeled with Alexa Fluor 488-conjugated anti-F4/80 antibodies and imaged by spinning disk confocal microscopy using brightfield and green fluorescent channels.

### Statistics

Normality and homoscedasticity were tested using the Shapiro-Wilk and Levene tests, respectively. A one-way ANOVA (analysis of variance) was used to test for statistical differences at the 0.05 level of significance. When the assumed conditions of normality and homogeneity of variance were not fulfilled, as in most cases, we used the non-parametric Mann-Whitney *U* test or Kruskal-Wallis one way analysis of variance on ranks (at the 0.05 level of significance). Statistical analyses were performed using Origin 2016 (OriginLab), and data are presented as box plots or mean ± standard error (s.e.m.).

## References

[1] Roland JT (2011). Rab GTPase-Myo5B complexes control membrane recycling and epithelial polarization. Proc Natl Acad Sci USA.

[2] Bahler M, Elfrink K, Hanley PJ, Thelen S, Xu Y (2011). Cellular functions of class IX myosins in epithelia and immune cells. Biochem Soc Trans.

[3] Berg JS, Cheney RE (2002). Myosin-X is an unconventional myosin that undergoes intrafilopodial motility. Nat Cell Biol.

[4] Kerber ML, Cheney RE (2011). Myosin-X: a MyTH-FERM myosin at the tips of filopodia. J Cell Sci.

[5] Wu L, Pan L, Wei Z, Zhang M (2011). Structure of MyTH4-FERM domains in myosin VIIa tail bound to cargo. Science.

[6] Belyantseva IA, Boger ET, Friedman TB (2003). Myosin XVa localizes to the tips of inner ear sensory cell stereocilia and is essential for staircase formation of the hair bundle. Proc Natl Acad Sci USA.

[7] Chen ZY (2001). Myosin-VIIb, a novel unconventional myosin, is a constituent of microvilli in transporting epithelia. Genomics.

[8] Weck ML, Crawley SW, Stone CR, Tyska MJ (2016). Myosin-7b Promotes Distal Tip Localization of the Intermicrovillar Adhesion Complex. Curr Biol.

[9] Yu IM (2017). Myosin 7 and its adaptors link cadherins to actin. Nature communications.

[10] Manor U (2011). Regulation of stereocilia length by myosin XVa and whirlin depends on the actin-regulatory protein Eps8. Curr Biol.

[11] Probst FJ (1999). & Camper, S. A. The role of mouse mutants in the identification of human hereditary hearing loss genes. Hearing research.

[12] Almagro S (2010). The motor protein myosin-X transports VE-cadherin along filopodia to allow the formation of early endothelial cell-cell contacts. Mol Cell Biol.

[13] Lai M (2015). Myosin X regulates neuronal radial migration through interacting with N-cadherin. Frontiers in cellular neuroscience.

[14] Zhang H (2004). Myosin-X provides a motor-based link between integrins and the cytoskeleton. Nat Cell Biol.

[15] Wei Z, Yan J, Lu Q, Pan L, Zhang M (2011). Cargo recognition mechanism of myosin X revealed by the structure of its tail MyTH4-FERM tandem in complex with the DCC P3 domain. Proc Natl Acad Sci USA.

[16] Sousa AD, Cheney RE (2005). Myosin-X: a molecular motor at the cell’s fingertips. Trends Cell Biol.

[17] Lu Q, Ye F, Wei Z, Wen Z, Zhang M (2012). Antiparallel coiled-coil-mediated dimerization of myosin X. Proc Natl Acad Sci USA.

[18] Lu Q, Yu J, Yan J, Wei Z, Zhang M (2011). Structural basis of the myosin X PH1(N)-PH2-PH1(C) tandem as a specific and acute cellular PI(3,4,5)P(3) sensor. Mol Biol Cell.

[19] Berg JS, Derfler BH, Pennisi CM, Corey DP, Cheney RE (2000). Myosin-X, a novel myosin with pleckstrin homology domains, associates with regions of dynamic actin. J Cell Sci.

[20] Weber KL, Sokac AM, Berg JS, Cheney RE, Bement WM (2004). A microtubule-binding myosin required for nuclear anchoring and spindle assembly. Nature.

[21] Pi X (2007). Sequential roles for myosin-X in BMP6-dependent filopodial extension, migration, and activation of BMP receptors. J Cell Biol.

[22] Singh SK, Abbas WA, Tobin DJ (2012). Bone morphogenetic proteins differentially regulate pigmentation in human skin cells. J Cell Sci.

[23] Zhu XJ (2007). Myosin X regulates netrin receptors and functions in axonal path-finding. Nat Cell Biol.

[24] Hwang YS, Luo T, Xu Y, Sargent TD (2009). Myosin-X is required for cranial neural crest cell migration in Xenopus laevis. Dev Dyn.

[25] Nie S, Kee Y, Bronner-Fraser M (2009). Myosin-X is critical for migratory ability of Xenopus cranial neural crest cells. Dev Biol.

[26] Cox D (2002). Myosin X is a downstream effector of PI(3)K during phagocytosis. Nat Cell Biol.

[27] Heimsath EG (2017). Myosin-X knockout is semi-lethal and demonstrates that myosin-X functions in neural tube closure, pigmentation, hyaloid vasculature regression, and filopodia formation. Scientific reports.

[28] Tokuo H, Bhawan J, Coluccio LM (2018). Myosin X is required for efficient melanoblast migration and melanoma initiation and metastasis. Scientific reports.

[29] Sousa AD, Berg JS, Robertson BW, Meeker RB, Cheney RE (2006). Myo10 in brain: developmental regulation, identification of a headless isoform and dynamics in neurons. J Cell Sci.

[30] Raines AN, Nagdas S, Kerber ML, Cheney RE (2012). Headless Myo10 is a negative regulator of full-length Myo10 and inhibits axon outgrowth in cortical neurons. J Biol Chem.

[31] Copp AJ, Greene ND (2013). Neural tube defects–disorders of neurulation and related embryonic processes. Wiley interdisciplinary reviews. Developmental biology.

[32] Umeki N (2011). Phospholipid-dependent regulation of the motor activity of myosin X. Nature structural & molecular biology.

[33] Gomez TM, Letourneau PC (2014). Actin dynamics in growth cone motility and navigation. Journal of neurochemistry.

[34] Fazeli A (1997). Phenotype of mice lacking functional Deleted in colorectal cancer (Dcc) gene. Nature.

[35] Serafini T (1996). Netrin-1 is required for commissural axon guidance in the developing vertebrate nervous system. Cell.

[36] Hirano Y (2011). Structural basis of cargo recognition by the myosin-X MyTH4-FERM domain. EMBO J.

[37] Liakath-Ali K (2014). Novel skin phenotypes revealed by a genome-wide mouse reverse genetic screen. Nature communications.

[38] Pingault V (2010). Review and update of mutations causing Waardenburg syndrome. Human mutation.

[39] Ghosh SK, Bandyopadhyay D, Ghosh A, Biswas SK, Mandal RK (2010). Waardenburg syndrome: a report of three cases. Indian journal of dermatology, venereology and leprology.

[40] Ritter MR (2005). Three-dimensional *in vivo* imaging of the mouse intraocular vasculature during development and disease. Investigative ophthalmology & visual science.

[41] Lobov IB (2005). WNT7b mediates macrophage-induced programmed cell death in patterning of the vasculature. Nature.

[42] Xu Q (2004). Vascular development in the retina and inner ear: control by Norrin and Frizzled-4, a high-affinity ligand-receptor pair. Cell.

[43] Kato M (2002). Cbfa1-independent decrease in osteoblast proliferation, osteopenia, and persistent embryonic eye vascularization in mice deficient in Lrp5, a Wnt coreceptor. J Cell Biol.

[44] Luhmann UF (2005). Role of the Norrie disease pseudoglioma gene in sprouting angiogenesis during development of the retinal vasculature. Investigative ophthalmology & visual science.

[45] Gerhardt H (2003). VEGF guides angiogenic sprouting utilizing endothelial tip cell filopodia. J Cell Biol.

[46] Horsthemke M (2017). Multiple roles of filopodial dynamics in particle capture and phagocytosis and phenotypes of Cdc42 and Myo10 deletion. J Biol Chem.

[47] de Haij S (2010). *In vivo* cytotoxicity of type I CD20 antibodies critically depends on Fc receptor ITAM signaling. Cancer research.

[48] Closse C, Dachary-Prigent J, Boisseau MR (1999). Phosphatidylserine-related adhesion of human erythrocytes to vascular endothelium. British journal of haematology.

[49] Hochreiter-Hufford A, Ravichandran KS (2013). Clearing the dead: apoptotic cell sensing, recognition, engulfment, and digestion. Cold Spring Harbor perspectives in biology.

[50] Copp AJ, Greene ND, Murdoch JN (2003). The genetic basis of mammalian neurulation. Nature reviews. Genetics.

[51] Liu KC, Jacobs DT, Dunn BD, Fanning AS, Cheney RE (2012). Myosin-X functions in polarized epithelial cells. Mol Biol Cell.

[52] Pyrgaki C, Trainor P, Hadjantonakis AK, Niswander L (2010). Dynamic imaging of mammalian neural tube closure. Dev Biol.

[53] Nikolopoulou E, Galea GL, Rolo A, Greene ND, Copp AJ (2017). Neural tube closure: cellular, molecular and biomechanical mechanisms. Development.

[54] Luo Y (2001). Rescuing the N-cadherin knockout by cardiac-specific expression of N- or E-cadherin. Development.

[55] Luo Y, High FA, Epstein JA, Radice GL (2006). N-cadherin is required for neural crest remodeling of the cardiac outflow tract. Dev Biol.

[56] Simoes-Costa M, Bronner ME (2015). Establishing neural crest identity: a gene regulatory recipe. Development.

[57] Ishii M (2012). A stable cranial neural crest cell line from mouse. Stem cells and development.

[58] Hu YF, Zhang ZJ, Sieber-Blum M (2006). An epidermal neural crest stem cell (EPI-NCSC) molecular signature. Stem Cells.

[59] Bondurand N, Southard-Smith EM (2016). Mouse models of Hirschsprung disease and other developmental disorders of the enteric nervous system: Old and new players. Dev Biol.

[60] Plouhinec JL (2014). Pax3 and Zic1 trigger the early neural crest gene regulatory network by the direct activation of multiple key neural crest specifiers. Dev Biol.

[61] Li A (2011). Rac1 drives melanoblast organization during mouse development by orchestrating pseudopod- driven motility and cell-cycle progression. Developmental cell.

[62] Woodham EF (2017). Coordination by Cdc42 of Actin, Contractility, and Adhesion for Melanoblast Movement in Mouse Skin. Curr Biol.

[63] Liakath-Ali K, Vancollie VE, Sequeira I, Lelliott CJ, Watt FM (2018). Myosin 10 is involved in murine pigmentation. Experimental dermatology.

[64] Bandyopadhyay A (2006). Genetic analysis of the roles of BMP2, BMP4, and BMP7 in limb patterning and skeletogenesis. PLoS Genet.

[65] Morello R (2008). Brachy-syndactyly caused by loss of Sfrp2 function. Journal of cellular physiology.

[66] Mukhopadhyay M (2001). Dickkopf1 is required for embryonic head induction and limb morphogenesis in the mouse. Developmental cell.

[67] Lindsten T (2000). The combined functions of proapoptotic Bcl-2 family members bak and bax are essential for normal development of multiple tissues. Molecular cell.

[68] Ren D (2010). BID, BIM, and PUMA are essential for activation of the BAX- and BAK-dependent cell death program. Science.

[69] Wood W (2000). Mesenchymal cells engulf and clear apoptotic footplate cells in macrophageless PU.1 null mouse embryos. Development.

[70] Ohlmann AV, Adamek E, Ohlmann A, Lutjen-Drecoll E (2004). Norrie gene product is necessary for regression of hyaloid vessels. Investigative ophthalmology & visual science.

[71] Wang Y (2012). Norrin/Frizzled4 signaling in retinal vascular development and blood brain barrier plasticity. Cell.

[72] Xia CH, Lu E, Zeng J, Gong X (2013). Deletion of LRP5 in VLDLR knockout mice inhibits retinal neovascularization. PLoS One.

[73] Korn C, Augustin HG (2015). Mechanisms of Vessel Pruning and Regression. Developmental cell.

[74] Stanganello E, Scholpp S (2016). Role of cytonemes in Wnt transport. J Cell Sci.

[75] Poche RA, Hsu CW, McElwee ML, Burns AR, Dickinson ME (2015). Macrophages engulf endothelial cell membrane particles preceding pupillary membrane capillary regression. Dev Biol.

[76] Dent EW (2007). Filopodia are required for cortical neurite initiation. Nat Cell Biol.

[77] Yamaguchi Y, Miura M (2015). Programmed Cell Death and Caspase Functions During Neural Development. Current topics in developmental biology.

[78] Plantard L (2010). PtdIns(3,4,5)P(3) is a regulator of myosin-X localization and filopodia formation. J Cell Sci.

[79] Jeong HW (2017). Transcriptional regulation of endothelial cell behavior during sprouting angiogenesis. Nature communications.

[80] Pitulescu ME, Schmidt I, Benedito R, Adams RH (2010). Inducible gene targeting in the neonatal vasculature and analysis of retinal angiogenesis in mice. Nature protocols.

[81] Zudaire E, Gambardella L, Kurcz C, Vermeren S (2011). A computational tool for quantitative analysis of vascular networks. PLoS One.

